# Characterization, comparison, and phylogenetic analyses of chloroplast genomes of *Euphorbia* species

**DOI:** 10.1038/s41598-024-66102-0

**Published:** 2024-07-04

**Authors:** Soo-Rang Lee, Ami Oh, Dong Chan Son

**Affiliations:** 1 Department of Biology Education, College of Education, Chosun University, Gwangju, 61452 Republic of Korea; 2 Division of Forest Biodiversity and Herbarium, Korea National Arboretum, Pocheon, 11186 Republic of Korea

**Keywords:** Chloroplast genome, Comparative analysis, *Euphorbia*, Intraspecific variation, Phylogenetic analysis, Phylogenetics, Comparative genomics, Genome evolution

## Abstract

The genus *Euphorbia* (Euphorbiaceae) has near-cosmopolitan distribution and serves as a significant resource for both ornamental and medicinal purposes. Despite its economic importance, *Euphorbia*'s taxonomy has long been challenged by the intricate nature of morphological traits exhibiting high levels of convergence. While molecular markers are essential for phylogenetic studies, their availability for *Euphorbi*a has been limited. To address this gap, we conducted comparative analyses focusing on the chloroplast (CP) genomes of nine *Euphorbia* species, incorporating three newly sequenced and annotated accessions. In addition, phylogenetic informativeness and nucleotide diversity were computed to identify candidate markers for phylogenetic analyses among closely related taxa in the genus. Our investigation revealed relatively conserved sizes and structures of CP genomes across the studied species, with notable interspecific variations observed primarily in non-coding regions and IR/SC borders. By leveraging phylogenetic informativeness and nucleotide diversity, we identified *rpoB* gene as the optimal candidate for species delimitation and shallow-level phylogenetic inference within the genus. Through this comprehensive analysis of CP genomes across multiple taxa, our study sheds light on the evolutionary dynamics and taxonomic intricacies of *Euphorbia*, offering valuable insights into its CP genome evolution and taxonomy.

## Introduction

*Euphorbia* L. (Euphorbiaceae), comprising about 2000 recognized species^1^, shows a near-cosmopolitan distribution, predominantly found in tropical, subtropical and temperate regions^2^. Many species within the genus are valued as ornamental and garden plants, while some taxa have been used historically employed for medicinal purposes, particularly in treating digestive system disorders, skin ailments, and infections^2^. Morphologically, the genus stands out for its distinctive inflorescence known as cyathium, resembling a bisexual flower^3–5^. The cyathium has a cup-shaped involucre that bears clusters of reduced male and female flowers^3,6^, serving as a taxonomically significant characteristic for the species identification in *Euphorbia*^7^. *Euphorbia* is renowned for its notable morphological diversity and various growth forms, ranging from small herbs, shrubs to large canopy trees^6,8^.

The taxonomy of *Euphorbia* has posed challenges due to a high degree of convergence in various characters, such as unifying structure of cyathium, succulence of stems, development of thorns, richness of species within the genus, and its worldwide distribution^9–11^. However, over the past decades, many phylogenetic studies have improved our understanding for the taxonomy of the genus. All molecular phylogenetic analyses to date support the monophyly of *Euphorbia*. These studies confirmed the division of *Euphorbia* into four monophyletic subclades: *Esula*, *Euphorbia*, *Chamaesyce,* and *Athymalus*^6,10,12,13^. These four subgenera exhibit clear distinctions in terms of distribution, morphology and growth form.

Subgenus *Esula* distributed in the temperate Northern Hemisphere, primarily comprises annual or perennial herbs and shows the least diversity in growth form and plant structure among the four subgenera^6,14^. Most species are characterized by well-developed leaves and a lack of stipules. The cyathia are arranged in cymose rays around a terminal cyathium, with these rays further divided into dichasial branches. The involucral glands are entire, crenate, or have horn-like but never petaloid appendages. Subgenus *Euphorbia* spanning the tropics and subtropics, showcases the greatest diversity in species richness and growth form^6^. The subgenus contains over 650 species and exhibits particularly high variation in cyathial morphology. Subgenus *Chamaesyce* encompasses most New World *Euphorbia* species, which are predominantly herbaceous and non-succulent^6,14^. Remarkably, it is the only plant lineage at or below the genus level known to exhibit all photosynthetic types: C3, C4, and CAM, alongside a C2 system that represents an early stage in the transition from C3 to C4 photosynthesis. Lastly, subg. *Athymalus* confined primarily to the Old World, particularly Africa, is characterized by the development of succulent stems and roots, typically featuring sympodial branching with terminal inflorescence^6,14,15^. Although it is the smallest of the four subgenera of *Euphorbia*, it exhibits considerable diversity in growth forms involving a trend toward increased succulence, marked by a shift of photosynthetic functions from the leaves to the stems and branches.

The chloroplast (CP) genomes of most land plants comprise a closed circular DNA molecule with a quadripartite structure including a large single-copy region (LSC), a small single-copy region (SSC), and a pair of inverted regions (IR) separating the two single-copy regions. In general, the length of the CP genome falls within the range of 120–210 kb^16,17^ with a few exceptions, such as *Cathaya argyrophylla* (107 kb)^17^. The CP genome harbors about 110–130 genes^17,18^ and its structure and organization of the CP genomes, including gene content and order, remain highly conserved relative to nuclear and mitochondrial genomes^19–22^. Due to its conserved quadripartite structure, absence of recombination, maternal inheritance, and moderate nucleotide substitution rate, the CP genome has become a widely employed tool for deciphering plant phylogeny and evolution^18,22–26^.

Several genetic markers derived from CP genome sequences, such as *matK*, *rbcL*, and *trnH*-*psbA*, have been widely used in phylogenetic inferences, species delimitation, and DNA barcoding^27–31^. While these markers have proven valuable in some cases, they often exhibit variations that are too subtle to adequately resolve phylogenetic relationships among taxa^25^. Recently, whole CP genome sequences have become a common tool for phylogenetic analyses, species delimitation, and population genetic studies^11,32^. The advent of Next Generation Sequencing (NGS) has enabled faster and more cost-effective sequencing of CP genomes^33^, leading to a rapid increase in the number of publicly available complete CP genome sequences^34^. With more phylogenetic information retained from the whole CP genome data, many phylogenetic and taxonomic riddles have been successfully resolved^26,35–38^. An illustrative example of this is the study where 83 genes derived from 86 complete CP genomes were employed to elucidate the phylogenetic relationships between clades within Eudicotyledoneae^36,39–41^. Meanwhile, in the case of *Euphorbia*, although a large number of complete CP genome sequences are currently available (n = 170 ~) in the GenBank database, only a handful of studies have focused on the comparative analyses of the CP genomes^11,42^. Especially, the level of variation in a plastid genome for a species, which could be informative in population genetic studies, remains largely explored.

In the present study, we aimed to characterize the CP genomes of *Euphorbia* in depth, using both comparative and phylogenetic approaches. The goals of our research were: (1) to characterize structure and pattern of diversity in CP genomes of *Euphorbia* taxa by validating and comparing formerly reported CP genomes including three newly sequenced CP genomes in the current study; (2) to investigate intraspecific variations among multiple CP genomes within a single species; (3) to identify molecular markers within CP genome that could serve as alternatives to whole CP genome data, particularly for inter- and infra-species level phylogenetic and/or species delimitation studies in *Euphorbia*. To address the goals, we conducted the sequencing, assembly, and annotation of CP genomes from three *Euphorbia* species belonging to the subgenus *Chamaesyce*. In addition, we incorporated data from 18 accessions representing 9 *Euphorbia* species along with three outgroup taxa to infer phylogenetic trees. Through the utilization of varying datasets, we sought to assess the practical utility of CP genome in advancing our understanding of evolutionary relationships within the genus *Euphorbia*.

## Results

### Chloroplast genome assembly and annotation

The genomic libraries for *E. nutans*, *E. humifusa*, and *E. maculata* generated about 8.23 million, 7.54 million, and 6.92 million high-quality 300 bp paired-end reads, respectively. Two different assembly methods showed nearly no difference in our analysis. Prior to the comparative analyses for the nine *Euphorbia* species, the annotations for all 18 accessions used in the study, including the three newly assembled samples, were closely examined to avoid potential errors. All 15 accessions retrieved from GenBank showed incorrect annotations, therefore, modifications were made to these annotations. For example, three accessions of *E. maculata* (NC_052745, MW496381, and OQ184027) were initially annotated with 129, 127, and 131 genes, respectively, indicating errors in their annotations. In our study, the gene count for these accessions was corrected to 132 following the reexamination results.

The annotation results showed that the three newly assembled accessions have the same coding sequence (CDS) and gene numbers as other accessions previously reported within the same species (Table 1), validating the accurate species identification and annotation of the new accessions. In addition, accession OM791345, initially labeled as *E. humifusa* in GenBank was excluded from our study. The decision was based on the inability to justify the species identification through our phylogenetic analyses, coupled with the observed identical length of the IR region compared to that of *E. thymifolia*. The reported accession likely corresponds to *E. thymifolia* rather than *E. humifusa*, although our data lacked a voucher specimen for morphological examination to conclusively confirm its identity.

**Table 1:** Information of accessions used and summary of chloroplast genome features for nine *Euphorbia* species.

Species (subgenus)	Accession no.	Plastome size (bp)	LSC (bp)	SSC (bp)	IR (bp)	No. genes	No. CDSs	No. tRNA	No. rRNA	GC %
*E. hirta *(*Chamaesyce*)	NC_058203	164,340	91,373	18,259	27,354	131	86	37	8	35.4
	OQ184032	163,808	90,705	18,503	27,300	131	86	37	8	35.4
	MW429224	164,773	90,880	18,485	27,704	131	86	37	8	35.3
*E. maculate *(*Chamaesyce*)	NC_052745	162,685	90,514	18,527	26,822	132	87	37	8	35.4
	OR189521	162,742	90,569	18,529	26,822	132	87	37	8	35.4
	MW496381	162,752	90,579	18,529	26,822	132	87	37	8	35.4
	OQ184027	162,727	90,556	18,527	26,822	132	87	37	8	35.4
*E. humifusa *(*Chamaesyce*)	OQ184028	163,602	91,468	18,446	26,835	131	86	37	8	35.3
	OR189520	163,610	91,495	18,445	26,835	131	86	37	8	35.3
*E. thymifolia *(*Chamaesyce*)	NC_062827	163,135	90,894	18,609	26,816	131	86	37	8	35.3
	OQ184030	163,153	90,959	18,562	26,816	131	86	37	8	35.3
*E. prostrate *(*Chamaesyce*)	ON631059	162,858	90,580	18,570	26,854	131	86	37	8	35.3
	OQ184029	162,868	90,587	18,573	26,854	131	86	37	8	35.3
*E. nutans *(*Chamaesyce*)	OQ871366	163,441	93,305	17,578	26,279	129	84	37	8	35.2
	NC_072939	162,841	93,312	17,621	25,954	129	84	37	8	35.3
*E. pekinensis* (*Esula*)	NC_058897	162,002	90,225	18,067	26,855	133	88	37	8	35.7
*E. tirucalli* (*Euphorbia*)	NC_042193	163,091	91,259	18,168	26,832	133	88	37	8	35.6
*E. smithii* (*Athymalus*)	MN646684	162,172	91,158	18,602	26,206	133	88	37	8	35.8

Number of genes and CDSs, here, includes pseudogenes.

### Features of the chloroplast genomes in *Euphorbia*

All CP genomes of the nine *Euphorbia* species in the present study display the typical quadripartite structure, comprising LSC, SSC, and a pair of IR regions. Lengths of the CP genomes ranged from 162,002 bp (*E. pekinensis*) to 164,773 bp (*E. hirta*). LSC regions varied from 90,225 bp (*E. pekinensis*) to 93,312 bp (*E. nutans*), while SSC regions ranged from 17,578 bp (*E. nutans*) to 18,609 bp (*E. thymifolia*). The sizes of IR regions varied from 25,954 bp (*E. nutans*) to 27,704 bp (*E. hirta*; Table 1). The number of genes within the CP genome ranged from 129 (*E. nutans*) to 133 (*E. pekinensis*, *E. tirucalli*, and *E. smithii*), with that of CDS varying between 84 (*E. nutans*) and 88 (*E. pekinensis*, *E. tirucalli*, and *E. smithii*; Table 1). Most species in the subgenus *Chamaesyce* shared 131 genes and 86 CDSs except for *E. nutans*, which lacked *rpl2* and *rps19* genes. *Euphorbia maculata* has 132 genes and 87 CDSs, including an additional pseudogene *rps16* (Tables 1, 2). The GC content ranged from 35.2% (*E. nutans*) to 35.8% (*E. smithii*). All nine *Euphorbia* species analyzed in our study contained 8 rRNAs and 37 tRNAs (Tables 1, 2). We identified gene losses or pseudogenes in all nine species. The pseudogene *rps16* was found only in *E. maculata*, *E. pekinensis*, *E. smithii*, and *E. tirucalli*, while the pseudogene *infA* was present in *E. pekinensis*, *E. smithii*, and *E. tirucalli*, with the other species lacking these two genes.

**Table 2:** Genes present in the chloroplast genomes of nine *Euphorbia* species.

Category	Group of genes	Name of genes
Self-replication	Large subunit of ribosomal proteins	***rpl2****, rpl14, rpl16, rpl20, rpl22, ****rpl23****, rpl33, rpl36*
	Small subunit of ribosomal proteins	*rps2, rps3, rps4, ****rps7****, rps8, rps11, ****rps12****, rps14, rps15, rps18, ****rps19***
	DNA-dependent RNA polymerase	*rpoA, rpoB, rpoC1, rpoC2*
	Ribosomal RNA genes	***rrn4.5, rrn5, rrn16, rrn23***
	Transfer RNA genes	***trnA-UGC****, trnC-GCA, trnD-GUC, trnE-UUC, trnF-GAA, trnfM-CAU, trnG-GCC, trnG-UCC, trnH-GUG, ****trnI-CAU****, ****trnI-GAU****, trnK-UUU, ****trnL-CAA****, trnL-UAA, trnL-UAG, trnM-CAU, ****trnN-GUU****, trnP-UGG, trnQ-UUG, ****trnR-ACG****, trnR-UCU, trnS-GCU, trnS-GGA, trnS-UGA, trnT-GGU, trnT-UGU, ****trnV-GAC****, trnV-UAC, trnW-CCA, trnY-GUA*
Photosynthesis	Photosystem I	*psaA, psaB, psaC, psaI, psaJ*
	Photosystem II	*psbA, psbB, psbC, psbD, psbE, psbF, psbH, psbI, psbJ, psbK, psbL, psbM, psbN, psbT, psbZ*
	NADH dehydrogenase	*ndhA, ****ndhB****, ndhC, ndhD, ndhE, ndhF, ndhG, ndhH, ndhI, ndhJ, ndhK*
	Large subunit of rubisco	*rbcL*
	Subunits of ATP synthase	*atpA, atpB, atpE, atpF, atpH, atpI*
	Cytochrome b6/f complex	*petA, petB, petD, petG, petL, petN*
Other genes	Maturase	*matK*
	Protease	*clpP*
	Envelope membrane protein	*cemA*
	Acetyl-CoA carboxylase	*accD*
	C-type cytochrome synthesis gene	*ccsA*
Unknown function		***ycf1, ycf2****, ycf3, ycf4*
Pseudogenes		*infA*^*a*^*, rps16*^*b*^*, ****ycf15***

Genes in bold indicate the genes duplicated in the IR regions.

^a^Absent in subg. *Chamaesyce*.

^b^Absent in subg. *Chamaesyce* except for *E. maculata.*

### Comparative genomic analysis in the nine *Euphorbia* species

**Figure 1:**
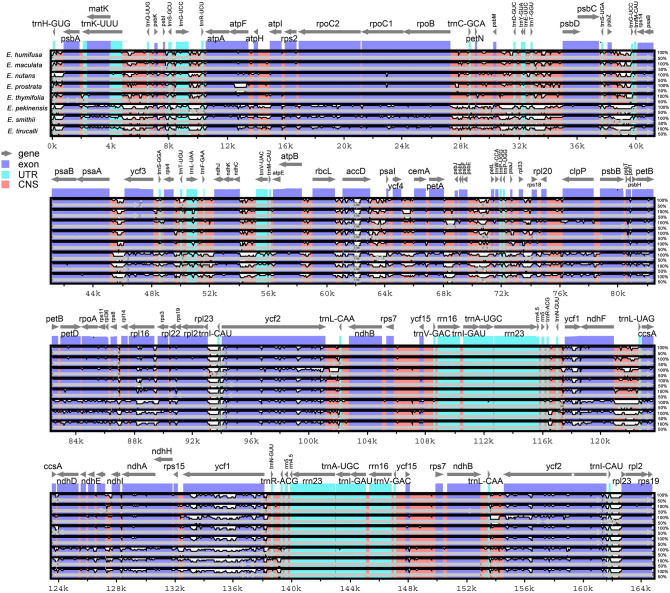
Plot of sequence divergence in chloroplast genomes among nine species of *Euphorbia* estimated from mVISTA. *Euphorbia hirta* was used as a reference to estimate the percent sequence identities. Pink and purple areas indicate non-coding regions and coding regions, respectively.

We assessed pairwise sequence divergence among the nine *Euphorbia* species using mVISTA, employing the annotation of *E. hirta* as a reference (Fig. 1). The results revealed considerable sequence divergence among the nine *Euphorbia* species, with coding regions displaying relatively low levels of sequence variation. Notably, three species, *E. pekinensis*, *E. smithii*, and *E. tirucalli*, which belong to the subgenus *Esula*, *Athymalus*, and *Euphorbia*, respectively, exhibited higher divergence compared to the other five species in the subg. *Chamaesyce* (Fig. 1).

**Figure 2:**
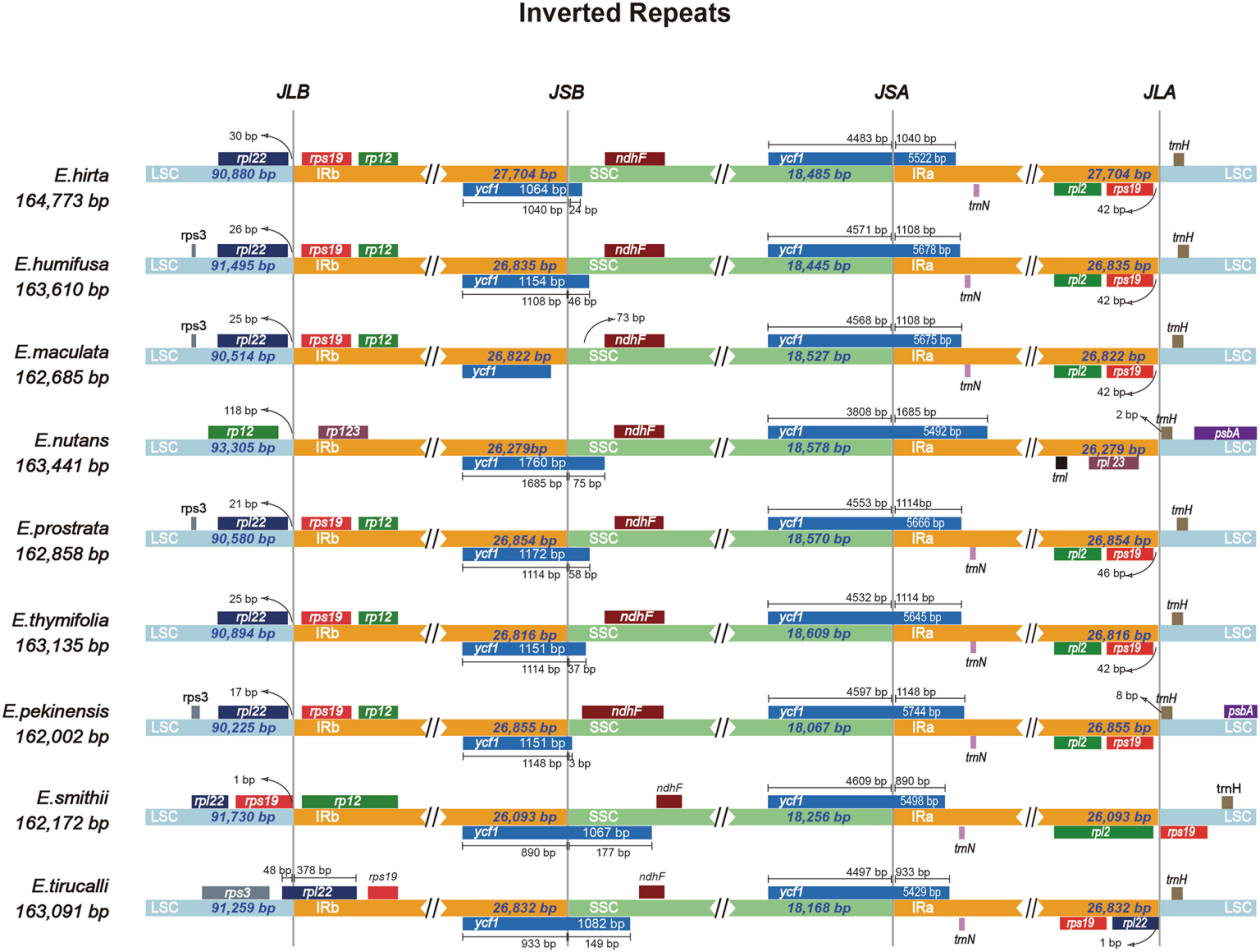
Comparison of the borders between the Large Single Copy (LSC), Inverted Repeat (IR), and Small Single Copy (SSC) regions among chloroplast genomes of nine *Euphorbia* species.

The IR/SC boundary regions were compared among the nine *Euphorbia* species (Fig. 2). Overall, the IR/SC boundary regions exhibited large variation, although the content and the coordinates of the genes across four borders (LSC/IRb, IRb/SSC, SSC/IRa, and IRa/LSC) exhibited some degree of similarity (Fig. 2). The LSC/IRb border was predominantly located between *rpl22* and *rps19* in most species, except for *E. nutans*, where it was positioned between *rpl2* and *rpl23* (Fig. 2). The difference in the border position correlates with the contraction of the IR region and the shorter length of IR (26,279 bp) in the species. In *E. tirucalli*, *rpl22* was situated at the same border, indicating an expansion of the IR region. Conversely, in *E. smithii*, the border was between *rps19* and *rpl2*, likely due to its shorter IR length (26,093 bp). The IRb/SSC border was generally located within *ycf1* across most species, although in two of four *E. maculate* accessions, *ycf1* was contained within the IRb region (Fig. 2). The SSC/IRa border was consistently found within *ycf1* in all species, despite significant variation in the lengths of *ycf1* genes among the nine species, ranging from 5429 to 5744 bp. Primarily, the IRa/LSC border was located between *rps19* and *trnH* across most species, although in *E. pekinensis*, *trnH* extended into IRa by 8 bp. In *E. smithii*, the border was between *rpl2* and *rps19*, while in *E. nutans*, it was between *rpl23* and *trnH*. Overall, the six species within the subg. *Chamaesyce* exhibited similar border structure, except for *E. nutans*, where the genes flanking the LSC/IRb and IRa/LSC borders differed from the other five species. In addition, distinct border structure differences were observed in *E. tirucalli* (subg. *Euphorbia*) and *E. smithii* (subg. *Athymalus*) compared to the six *Chamaesyce* species in the LSC/IRb and IRb/LSC regions. However, *E. pekinensis* (subg. *Esula*) showed a nearly identical border structure to the six *Chamaesyce* species (Fig. 2).

### Comparison between interspecific and intraspecific genomic variation

**Figure 3:**
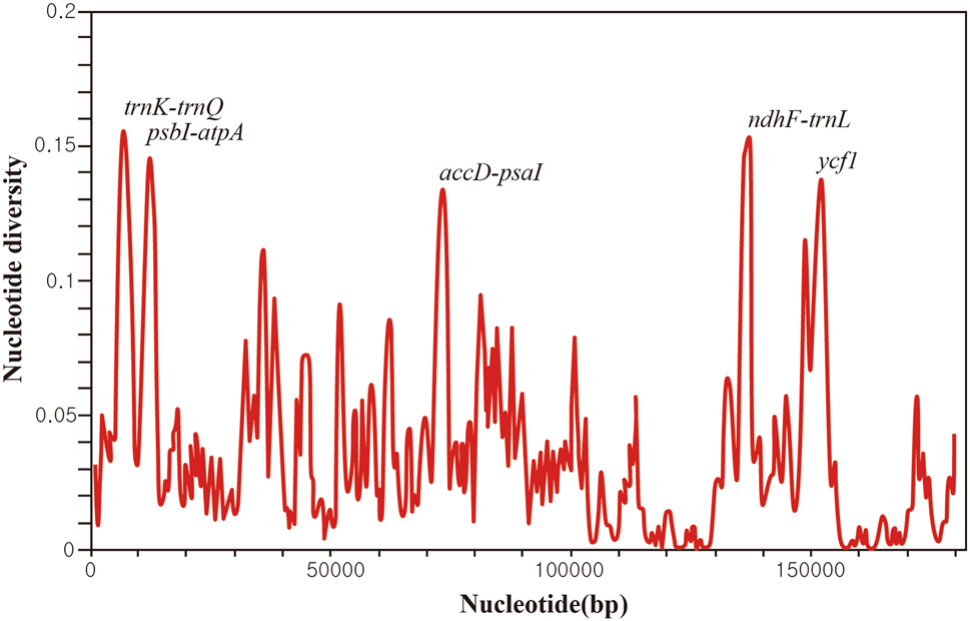
Nucleotide diversity plot estimated among the chloroplast genomes of nine *Euphorbia* species.

**Figure 4:**
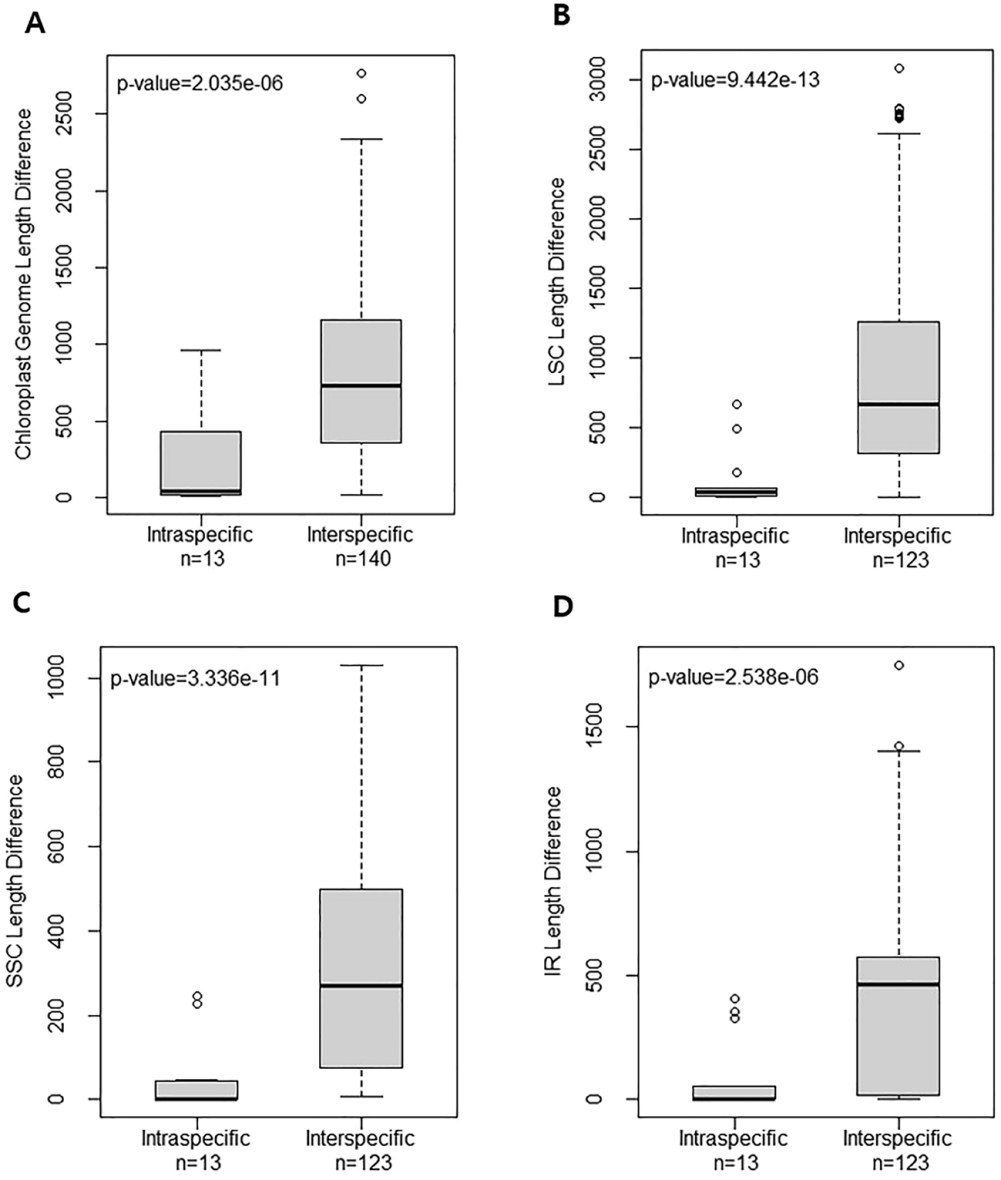
Bar plots illustrating inter- and intra-specific variations in genome size. (**A**) Variation in complete CP genome length; (**B**) Variation in Large Single Copy (LSC) region length; (**C**) Variation in Small Single Copy (SSC) region length; and (**D**) Variation in Inverted Repeat (IR) region length. The lines within the boxes represent the mean values. Error bars, denoted by vertical dotted lines, indicate standard deviation.

The interspecific genomic variation and the intraspecific genomic variation were compared across nine *Euphorbia* species (Table 1; Figs. 3, 4; Figs. S1–S3). During the comparison of genome sizes, we observed length variations among different individuals within the same species. In contrast, the number of CDSs and genes remained identical within each species used in the analysis. We noted no intraspecific variation in IR lengths for nearly half of the nine species (*E. maculata*, *E. thymifolia*, *E. prostrata* and *E. humifusa*; Table 1). However, the lengths of LSC and SSC regions differed among the individuals within a species except for one species, *E. maculata*, where two individuals within the species shared the same SSC length (Table 1). The GC contents were identical within a species except for *E. nutans* and *E. hirta*, where slightly different GC contents were detected (Table 1).

The mVISTA results revealed significantly low intraspecific sequence divergence for *E. humifusa*, *E. prostrata*, *E. thymifolia*, and *E. nutans*, with only minor variations in non-coding regions (Fig. S1). In contrast, *E. maculata* exhibited slightly higher variation compared to the four aforementioned species. Notably, *E. hirta* showed much greater sequence polymorphism than the other species, with noticeable divergences observed in both coding and non-coding regions (Fig. S1). Regarding gene order and distances from the inverted repeat (IR) or single-copy (SC) borders within a species, *E. humifusa*, *E. prostrata*, and *E. thymifolia* exhibited identical patterns (Fig. S2). Slight intraspecific variation in gene positions from the borders was observed in *E. nutans*, *E. hirta*, and *E. maculata* (Fig. S2).

We investigated genomic regions with high nucleotide diversity (pi) and compared the interspecific pi values to the intraspecific values calculated in six *Chamaesyce* species. Highly polymorphic regions differed between the inter- and intra-specific levels (Figs. 3, S3). In the interspecific pi calculations (mean = 0.028), the top five regions with the highest pi values were *trnK*-*trnQ*, *accD*-*psaI*, *ndhF*-*trnL*-UAG, *psbI*-*atpA*, and *ycf1* (Fig. 3). However, most of these regions were not identified as regions with high pi in intraspecific pi calculations (Fig. S3). Among the approximately130 genes in the CP genome, *ycf1* consistently showed high pi values at both inter- and intra-specific levels, particularly in *E. hirta*, *E. thymifolia*, *E. nutans*, and *E. maculata*. Furthermore, several loci such as *trnK*-*trnQ* (*E. humifusa*), *accD-psaI* (*E. maculata*)*, ccsA-ndhD* (*E. thymifolia*) exhibited high pi values within a species, although the magnitude varied widely across species (Fig. S3). Notably, we found nearly zero intraspecific sequence divergence for most genes, except for a few genes with high pi values in most species analyzed. However, the intraspecific pi values for regions with high pi values such as *accD*, *rpl23*-*ycf2* and *ycf1* were 10 times greater in *E. hirta* compared to the remaining species (Fig. S3). Moreover, even for loci with low to moderate pi values, the values were much greater than those estimated for the remaining species. Additionally, we compared the interspecific genome size variation to intraspecific variation and revealed that the former was significantly larger than the latter in all four regions of the *Euphorbia* CP genome (*P* < 0.05; Fig. 4).

### Phylogenetic analysis

**Figure 5:**
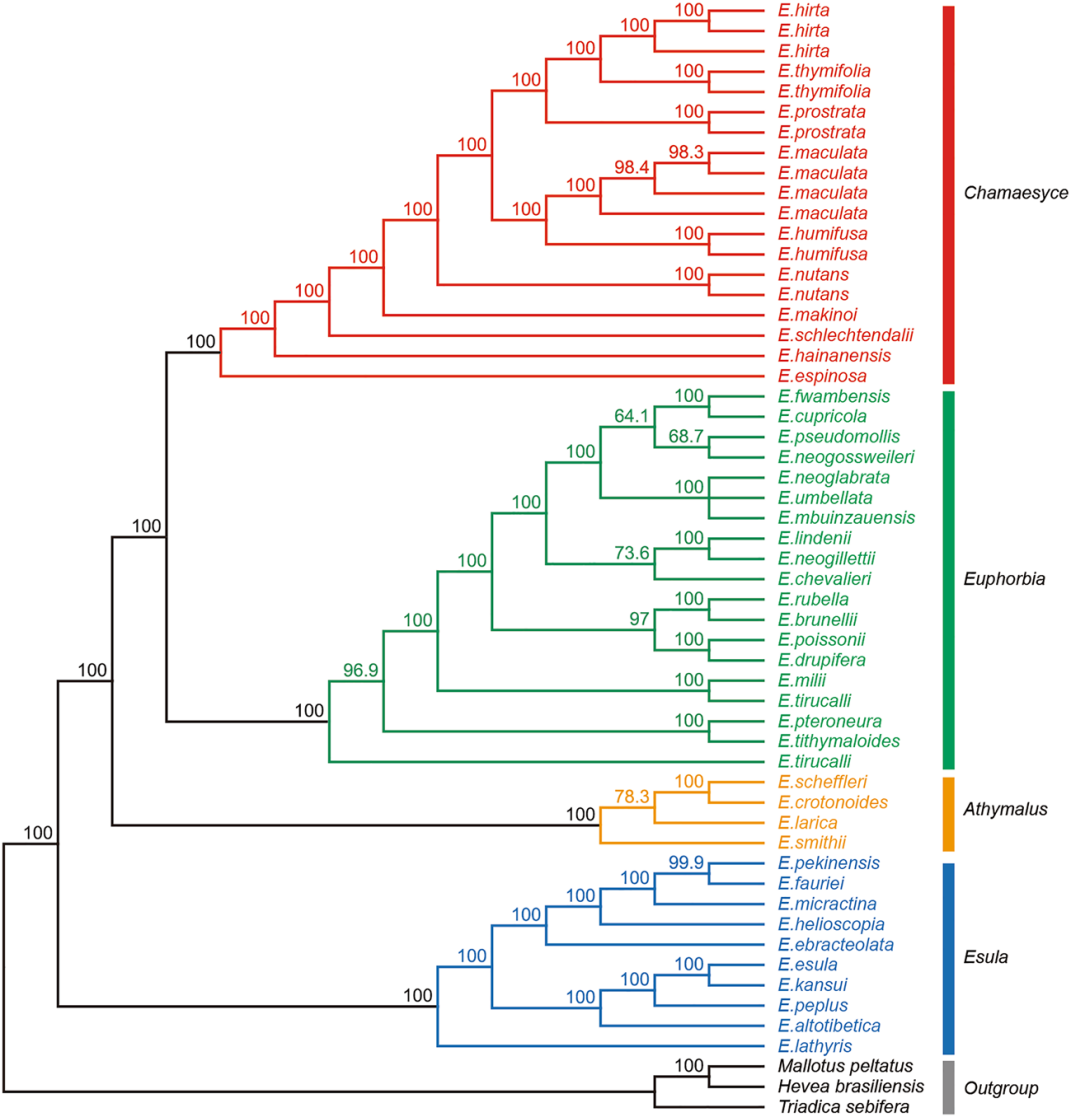
Phylogenetic relationships among 42 *Euphorbia* species inferred from complete CP genomes using the Maximum Likelihood (ML) method. Bootstrap values are indicated at branch nodes. The four colors represent each of the four subgenera in *Euphorbia*.

We inferred phylogenetic relationship in the genus *Euphorbia*, encompassing 42 species across four subgenera: *Chamaesyce* (10 species), *Euphorbia* (18 species), *Athymalus* (4 species), *Esula* (10 species). Our analysis utilized complete chloroplast genomes from 55 accessions, including the nine *Euphorbia* species under investigation and three outgroup species (Table S1). The resulting ML tree strongly supported monophyly of all four subgenera, with robust bootstrap values exceeding 99.9% (Fig. 5). Specifically, subgenus *Chamaesyce* emerged as a sister group to subgenus *Euphorbia*, while subgenus *Esula* occupied the basal position within the genus *Euphorbia*. Our phylogenetic analysis revealed clear species delimitation patterns, except for *E. tirucalli* (Fig. 5). Within *E. tirucalli*, the two accessions failed to form a monophyletic clade, suggesting potential ambiguity in their taxonomic classification (Fig. 5). The Bayesian tree generated using the same complete CP genomes showed a nearly identical topology to the ML tree, rendering further Bayesian Inference unnecessary for subsequent analyses (Fig. S4).

**Figure 6:**
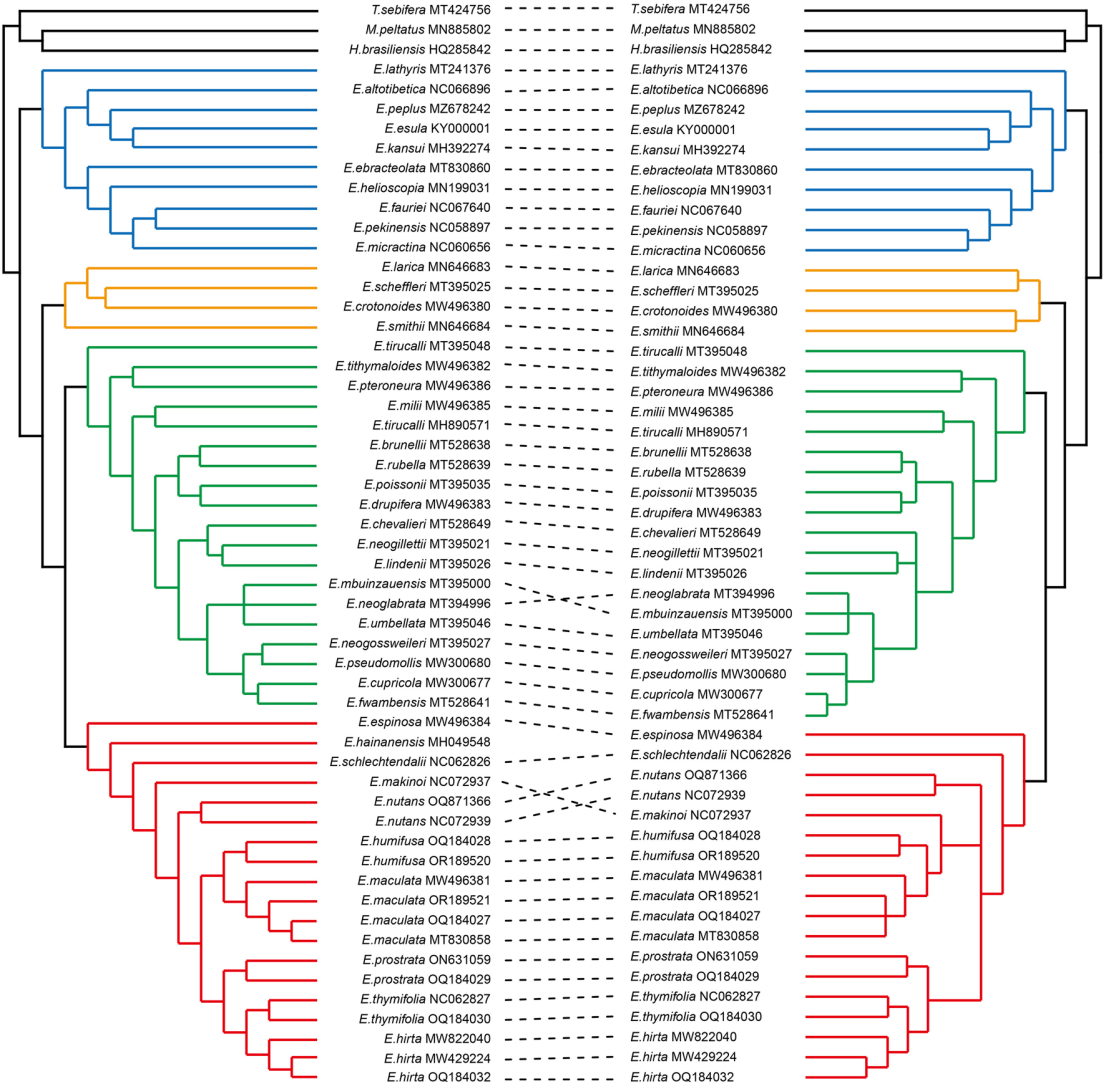
Phylogenetic relationships among *Euphorbia* species based on the rpoB gene (right) and complete CP genomes (left), inferred using the Maximum Likelihood (ML) method. Dashed lines connect identical samples in both trees. The four colors represent each of the four subgenera in *Euphorbia*: Red for subg. *Chamaesyce*, Green for subg. *Euphorbia*, Orange for subg. *Athymalus*, and Blue for subg. *Esula*.

We identified specific loci within the CP genome of *Euphorbia* that are well-suited for phylogenetic analysis and species delimitation. Five regions exhibiting the highest interspecific pi values, *trnK*-*trnQ*, *accD*-*psaI*, *ndhF*-*trnL*-UAG, *psbI*-*atpA*, and *ycf1*, were selected for further phylogenetic analyses. In addition, phylogenetic informativeness (PI) was computed for all CDSs to identify an additional set of informative loci for the phylogenetic inference in *Euphorbia*. Our PI computation highlighted 10 genes—*ycf1*, *ycf2*, *rpoC2*, *ndhF*, *matK*, *rpoB*, *accD*, *clpP*, *ndhD*, and *rpoC1*—with notably high PI values (Fig. S5). Given that both the five high pi regions and the ten high PI regions encompassed the *ycf1* gene, a total of 14 loci were individually employed to construct phylogenetic trees, with their tree topologies compared to that of the complete plastome tree. To identify the most informative loci combination, we explored all possible combinations of the three genes—*rpoB*, *rpoC1* and *rpoC2*—which individually exhibited the highest congruence with the reference tree (TreeDist values = 0.079, 0.097 and 0.101, respectively; Table S2). Among the combinations tested, the *rpoB* gene alone displayed the highest congruence level with the complete plastome tree, suggesting its efficacy as a singular marker for molecular diagnosis in *Euphorbia* (Fig. 6; Table S2). The concatenation of the *rpoB* and *rpoC2* genes yielded the second highest congruence with the reference tree (TreeDist = 0.085), followed by the concatenation of all three genes (TreeDist = 0.09), suggesting the potential utility of these markers combinations for the phylogenetic analyses in *Euphorbia* (Table S2).

## Discussion

*Euphorbia*, one of the largest genera in angiosperms with over 2000 species, has been a focal point of taxonomic disputes owing to high morphological variability and worldwide distribution^9–11^. In our study, we conducted a comparative analysis of the chloroplast (CP) genomes among nine *Euphorbia* species (18 accessions) including three newly collected, sequenced, and annotated *Euphorbia* accessions. Through comparative genomic analyses, we identified both inter- and intra-specific genome variations and discerned dozens of potential molecular markers suitable for phylogenetic inferences at various taxonomic levels—generic, inter-specific, and intra-specific. Our study revealed comparable variations in gene number, GC content, and the lengths of the genome and its substructures in relation to previously reported findings^11,42^. Notably, a study of Wei et al.^11^ observed a 42 kb variation in plastome length between two *Euphorbia* species, attributed to the near-complete loss of the inverted repeat (IR) region in *E. neogillettii*, a rare occurrence. The substantial genomic variation observed in our study may signify complex evolutionary events such as lineage divergence and introgression, as proposed by Wei et al.^11^ (Table 1).

Although the overall genomic structure of the CP characterized in our study was consistent with the previously reported CP genome structure^11,42^, we observed certain discrepancies among different studies^43–48^. For instance, the number of genes observed in our study differed from the ones reported in previous studies (Table 1). Specifically, we found 133 genes in *E. smithii* and 132 genes in *E. maculata* including pseudogenes, whereas previous studies reported 129–130 genes in *E. smithii* including the pseudogenes^11^ and 132 genes in *E. smithii*^42^. Additionally, the number of coding sequences (CDSs) and transfer RNA (tRNA) genes examined in our data varied from those reported in other studies^11,42^ (Table 1). The incongruence of genomic features may partly be attributed to the high CP genomic variation exhibited even within a single species in *Euphorbia*. Size variation may also be attributed to gene loss in certain accessions used in previous research. Gene loss is recognized as a significant driving mechanism of genomic variation in angiosperms^16–18^. The most common losses from the chloroplast genome are often due to gene transfer to the nucleus^16–18^. However, alternatively, the discrepancies observed between our study and previous studies may result from different annotation methods.

Three pseudogenes (*rps16*, *infA*, and *ycf15*) were identified in our study (Table 2). Of these, *rps16* and *infA* were present in *E. pekinensis*, *E. smithii*, and *E. tirucalli*, but were completely lost from most *Chamaesyce* species (Table 2). Previous CP genome studies have reported gene loss and pseudogenization of *rps16* not only in most *Euphorbia* species but also in other taxa within Euphorbiaceae, suggesting that *rps16* gene loss/pseudogenization likely occurred long before divergence of the genus *Euphorbia*^11,49^. *Rps16* plays a critical role in the assembly of the 30S subunit^38^ and cell viability in *Escherichia coli*^50^. Given its importance in plant survival, it is not surprising that the gene is present in the CP genomes of most angiosperms^51^. However, the gene is lost in many families of Malpighiales including Euphorbiaceae^11,49,52,53^, suggesting that gene loss might have contributed to the diversification of Malpighiales. In our data, *rps16* was completely lost or pseudogenized in all nine species, consistent with the previous findings. In contrast, *infA* (encoding for translation initiation factor 1^54^) is repeatedly lost in CP genomes of angiosperms such as many Rosid species^55^. There was no *infA* in subg. *Chamaesyce* indicating that the complete loss of *infA* likely occurred prior to the divergence of the subgenus. *Ycf15* was pseudogenized in *E. prostrata*, *E. thymifolia*, *E. pekinensis*, *E. smithii*, *E. tirucalli* (138 bp) and *E. hirta* (195 bp; Table 2). The gene is disabled or completely lost in many angiosperms, although intact gene copies are detected in some species^56^. In our study, a pair of *ycf15* was located within the IR region for all nine species. Interestingly, the position and size of the genes varied across the nine species, partly due to size reduction through pseudogenization.

Expansion and contraction of IR regions are common phenomena in CP genome, crucial for comprehending CP genome evolution and diversity^57–60^. Large changes in CP genome size and structure often correlate with IR contraction and expansion. In a previous study of Wei et al.^11^, remarkable IR region expansions were observed in *E. tithymaloides* and *E. schlechtendalii* alongside IR contraction in *E. neogillettii*. In our study, we noted significantly greater expansion/contraction of IR regions within the nine *Euphorbia* species compared to those reported in other angiosperm CP genomes (Fig. 2)^18,22,60,61^. For example, *E. smithii* and *E. nutans* exhibited IR contractions of approximately 700 bp and 550 bp IR, respectively, notably larger than in other taxa, such as *Hosta*, where IR contraction within the genus was less than 20 bp^61^. Furthermore, our data revealed the insertion of *ycf1* gene on the IR/SC borders in all nine species (Fig. 2). *Ycf1*, known as the second largest gene in the CP genome, plays a critical role in plant viability^62^. Pseudogenization of *ycf1* on the IR/SC border has been reported in several studies, indicating a tight link between the IR expansion/contraction and *ycf1* pseudogenization^60,63,64^. Overall, the substantial expansion and contraction of IR regions observed herein may be a major factor determining CP genome structure variation in *Euphorbia*. Therefore, it warrants further attention to understand CP genome evolution and the putative mechanisms driving diversification within the genus.

With the advent of Next Generation Sequencing, there has been a rapid increase in the number of CP genomes sequenced and comparative CP genome, with over 57,000 complete CP genome sequences available in GenBank (https://www.ncbi.nlm.nih.gov^59^). However, while most studies have focused on interspecific CP genome comparisons, research on intraspecific level genome comparisons is scarce. Our study represents the first examination of intraspecific CP genome variation and its comparison to the interspecific variation within the genus *Euphorbia*. As expected, the number and order of the genes, as well as the size and structure of the whole genome, were highly conserved within a species (Table 1; Figs S1, S2). Although the intraspecific variation in sequence divergence, measured by pi, was much lower than interspecific pi, certain loci exhibited high intraspecific pi (Figs. 3, S3). For instance, pi values for more than five loci computed in *E. hirta* were nearly comparable to the average interspecific pi among the six species (Figs. 3, S3). This finding was particularly noteworthy considering the limited number of intraspecific level samples used for pi estimation (Table 1). It is important to note that the majority of this high divergence stemmed from just one of the three *E. hirta* accessions (Fig. S1). Consequently, this heightened divergence could potentially be attributed to misidentification of that particular accession. Should this accession be erroneously labeled as *E. hirta*, it would likely exhibit substantial divergence from the other two accessions. Nevertheless, our phylogenetic analysis reveals that all three accessions are grouped together in a clade (Fig. 5). Alternatively, considering that *E. hirta* currently boasts a global distribution and has recently colonized vast regions of the world, it's plausible that it has been subjected to diverse selective pressures stemming from varying ecologies and environments. The adaptation to these diverse ecological and environmental conditions may have led to large sequence variation observed herein. However, again, this perspective should be taken with caution considering the similar worldwide distributions of other *Euphorbia* species, such as *E. prostrata*^65^, which shows intraspecific sequence variation of zero in our analysis. Our observation may be just due to sampling of three *E. hirta* accessions that are accidentally highly distant from the others, both geographically and molecularly. If more intraspecific samples of *E. hirta* are added in the future studies, we may expect to see higher pi values for more loci. While the pi values observed in the remaining four *Chamaesyce* species were notably lower than those in *E. hirta*, our analysis still revealed several loci with relatively high pi values (Fig. S3). These loci represent promising candidates for molecular markers in future population genetic studies and shallow-level phylogenetic research among closely related taxa.

Previous molecular phylogenetic studies on *Euphorbia* have consistently reported the monophyly of the genus, identifying four well-defined subclades, now recognized as four subgenera: *Esula*, *Euphorbia*, *Chamaesyce*, and *Athymalus*^10,12,14,66^. Consistent with these findings, our phylogeny, based on the whole CP genomes of 42 *Euphorbia* species, also recognized the four subgenera forming monophyletic groups (Fig. 5). Undoubtedly, reconstructing a phylogeny from whole CP genome is one of the most effective methods to resolve evolutionary relationships between taxa^60,67,68^. However, the assembly of many CP genomes is not cost effective. Therefore, in this study, we aimed to identify molecular markers within the CP genome that could infer a well-resolved phylogeny comparable to the whole CP genome data in the genus *Euphorbia*. Among the various combinations of the 14 loci, including both high pi regions and high PI regions, *rpoB* gene individually displayed the most congruent tree topology with the complete CP tree. In the phylogenetic tree constructed with *rpoB*, where the monophyly of the four subgenera was clearly supported, only minor differences in topology were observed compared to the complete CP tree (Fig. 6). These results suggest that the *rpoB* gene can be highly effective in the phylogenetic study of *Euphorbia*, both individually and potentially in combinations with other loci. Additionally, considering the significantly high congruence levels of the different combinations of *rpoB* with other high congruence genes such as *rpoC1* and *rpoC2*, *rpoC1* and *rpoC2* can also be employed for phylogenetic inference in *Euphorbia*. In addition, other loci with relatively low Tree Distance values, such as *matK*, *ndhF*, *psbI-atpA* and *ycf1*, can be candidates for promising molecular markers (Table S2). Indeed, *matK*, *ndhF* and *ycf1* have been previously identified or utilized as useful markers in phylogenetic studies of *Euphorbia*^11,12,42^. These findings suggest that both high pi and PI loci can serve as alternatives to the complete CP genome in phylogenetic analyses of *Euphorbia*, at a much lower cost. Future phylogenetic studies on *Euphorbia* may refer to our results when adopting a limited number of loci in the CP genome with high phylogenetic utility. Taken together, our multifaceted approaches collectively demonstrate that the CP genome, both as a whole and as partial fragments, can be an important tool for delimiting species and resolving phylogenetic relationships within the genus *Euphorbia*.

## Conclusion

Our study reveals that the size and the structure of CP genomes for the nine *Euphorbia* species are relatively conserved, despite clear interspecific genome variations. Sequence variations among these species are primarily observed in non-coding regions, with some species exhibiting events of IR contraction/expansion. As expected, intraspecific genome variation was significantly lower than interspecific variation. Our phylogenetic analysis aligns with previous findings on the chloroplast genome of *Euphorbia*, where each of the four subgenera formed a monophyletic clade and the relationships between these subgenera were highly consistent. Notably, the loci with high pi or PI values, identified in our analyses, can serve as effective molecular markers, offering alternatives to the complete CP genome sequencing. In conclusion, the CP genome of *Euphorbia* emerges as a valuable tool for understanding the phylogenetic relationships, taxonomy, and evolution of *Euphorbia* species. The relatively conserved nature, coupled with the utility of specific loci as molecular markers, underscores its importance in elucidating the evolutionary dynamics within this diverse genus.

## Methods

### Plant materials, DNA extraction, and sequencing

Three *Euphorbia* species, *Euphorbia nutans*, *E. humifusa*, and *E. maculata*, were collected and sequenced for the CP genome assembly and comparative analysis. *Euphorbia nutans* and *E. maculata* were collected from Mt. Gariwang, Jeongseon-gun (37° 25′ 27.12″ N, 128° 34′ 27.84″ E), and *E. humifusa* from Is. Bigeum, Sinan-gun (34° 45′ 6.84″ N, 125° 54′ 15.48″ E) in Korea. Collection of plant material complies with relevant institutional, national, and international guidelines and legislation. The identification of the plant material was undertaken by Dr. Dong-Chan Son. All total genomic DNAs were extracted from the silica-dried leaves using DNeasy Plant Mini Kit (Qiagen, Hilden, Germany), following manufacturer’s protocol. The quality of DNA was visually checked by electrophoresis on a 1% agarose gel. Library preparation and sequencing were performed by Macrogen Co. (Seoul, Korea). The sequencing was performed on an Illumina Miseq platform (Illumina, San Diego, CA). The voucher specimens for the three species were deposited in the herbarium of the Korean National Arboretum (KH) with the numbers of KHB1644876 (*E. humifusa*), KHB1644877 (*E. maculata*), and KHB1644878 (*E. nutans*). The CP genome sequences of 15 accessions from 9 *Euphorbia* species were downloaded from NCBI for the analyses (Table S1).

### Genome assembly and annotation

We employed two different approaches to assemble the chloroplast (CP) genomes of three newly sequenced species, *E. nutans*, *E. humifusa*, and *E.* maculata. Initially, we conducted a de novo assembly using NOVOPlasty v. 4.3.3^69^ (http://github.com/ndierckx/NOVOPlasty) utilizing the rbcL gene sequence of *E. maculata* (OQ184027) as a seed sequence. In addition, we used three reference genomes, *E. nutans* (NC_072939), *E. humifusa* (OQ184028), and *E. maculata* (OQ184027) and assembled the three newly sequenced genomes in Geneious v. 2022.0.1 (http://www.geneious.com). For the Geneious assembly, we filtered the raw reads using the 'Trim using BBDuk' function, removing adapters, low-quality bases (Q < 20), and short reads with a length < 10 bp. The remaining high-quality reads were then assembled using the 'Map to reference' function.

The assembled CP genomes were annotated using both ‘annotate from database’ function in Geneious and the online annotation tool GeSeq^70^. The annotation results were manually re-examined using Geneious to avoid potential annotation errors. The annotated CP genome sequences of *E. nutans*, *E. humifusa*, and *E. maculata* were prepared with GB2sequin^71^ and deposited into GenBank database (Accession No. OQ871366; OR189520; OR189521 respectively). While there are now over 170 CP genome sequences available for the genus *Euphorbia* in GenBank, many of these data are inaccurately annotated. Therefore, we meticulously reviewed and corrected any annotation errors identified in 15 accessions retrieved from GenBank (Table S1) using GeSeq, Geneious, and NCBI-BLAST^72^.

### Comparative chloroplast genome analysis

In our comparative plastome study, the genome sequence, structure, and gene organization were compared among 18 accessions of nine *Euphorbia* species using Geneious. For pairwise sequence divergence analysis among the *Euphorbia* accessions, mVISTA (http://genome.lbl.gov/vista/mvista/submit.shtml) was used with Shuffle-LAGAN alignment mode. *Euphorbia hirta* was used as a reference in mVISTA analysis. In addition, genes on the boundaries of LSC, IR, and SSC junction sites and expansion or contraction of the IR regions were compared among the accessions and visualized using the online program IRscope (http://irscope.shinyapps.io/irapp)^73^. For both pairwise sequence divergence analysis and boundary comparison, interspecific variation was analyzed using 9 accessions from nine species (one accession from each species). Intraspecific variation was analyzed for six species where the number of accessions used ranged from two to four (two accessions for *E. humifusa*, *E. thymifolia*, *E. prostrata*, and *E. nutans*; three accessions for *E. hirta*; four accessions for *E. maculata*; Table 1).

To compare intraspecific and interspecific genome size variations, both genome size differences among nine species and among the available accessions within each 6 species (*E. humifusa*, *E.thymifolia*, *E. prostrata*, *E. nutans*, *E. hirta*, and *E. maculata*) were calculated. In addition to the length variation of complete CP genome, the variations in LSC, SSC, and IR regions were analyzed. The length variation comparisons were visualized as a box plot in R v. 4.1.2^74^. T-test was conducted to confirm the significance of the difference between intraspecific and interspecific genome size variations using R.

For the comparative analysis, we also examined the nucleotide diversity using sliding window analysis in DnaSP v. 6.0^75^. The accessions from *Euphorbia* species were aligned using MAFFT v. 7.450^76^, and the step size was set to 200 bp and the window size to 600 bp in DnaSP. The loci with high nucleotide diversity (pi) values were compared between the interspecific pi calculation including nine accessions of different species and the intraspecific pi calculation including all the available accessions within a single species. Five loci with the highest nucleotide diversity (pi) from each calculation were selected and compared between the intraspecific pi and interspecific pi estimation.

### Phylogenetic analysis

To resolve the phylogenetic positions of nine *Euphorbia* species within the genus and understand the utility of CP genome in the phylogenetic study, we inferred a phylogeny with complete CP genome data. The complete CP genome sequences of additional 33 *Euphorbia* species and the three outgroups belonging to the family Euphorbiaceae, that are *Hevea basiliensis*, *Mallotus peltatus*, and *Triadica sebifera*, were downloaded from GenBank (Table S1). The sequences were aligned using MAFFT with the default parameters, and the phylogenetic inferences were performed using Maximum Likelihood (ML) method in Geneious. ML tree was inferred with GTR GAMMA nucleotide substitution model in RAxML v. 8.2.11^77^ with 1000 rapid bootstrap replicates for node support. ‘Consensus Tree Builder’ function was used to create a consensus tree from 1000 ML trees generated. We used 50% majority rule for the generation of consensus tree. In addition, we inferred another phylogenetic tree using Bayesian Inference (BI) method in Geneious with the same alignment as that of ML tree. BI tree was inferred with GTR GAMMA model in MrBayes v. 3.2.6^78^ with MCMC chain length of 100,000 generations, sampling frequency of 100 generations, and burn-in length of 10,000 generations.

To identify chloroplast (CP) genes suitable as alternatives to the whole plastome dataset for constructing phylogenetic trees, particularly at inter- and infra-species levels, we conducted phylogenetic analyses using selected genes with high polymorphism (pi) or phylogenetic informativeness (PI). Regions exhibiting congruence with the plastome tree were deemed viable alternatives. Firstly, we assessed phylogenetic informativeness (PI) for each coding sequence (CDS) using the PhyDesign web application (http://phydesign.townsend.yale.edu)^79^. HyPhy^80^, a third-party program, was utilized to estimate site rates under various time-reversible models, employing the net-profile approach. Subsequently, after inputting the alignment and tree to PhyDesign, HyPhy was employed to calculate evolutionary (substitution) rates for each alignment site, generating relevant data for phylogenetic informativeness.

We selected ten regions with high PI and five loci with high nucleotide diversity (pi) for subsequent phylogenetic analyses. Fourteen loci in total were chosen for further analyses, with some overlap between the high PI and high pi sets. Each of these 14 loci was used individually to construct phylogenetic trees, and their topologies were compared to those of complete CP genome trees to assess utility. Among the 14 loci, three displayed the highest congruence levels with the complete plastome tree. Subsequently, all possible combinations of these three regions were tested for congruence with the reference to identify the most useful marker combination. TreeDist, an R script, was employed to calculate generalized Robinson-Foulds distances, comparing bipartitions between trees to evaluate congruence. In cases where accessions had missing data for the locus used in the phylogenetic analysis, those accessions were excluded from that particular analysis. Consequently, each phylogenetic tree generated in our study includes a varying number of accessions (Table S1).

### Supplementary Information

Supplementary Information.

## Data Availability

The datasets generated and/or analyzed during the current study are available in GenBank. GenBank accession numbers are OQ871366 (*E. nutans*, https://www.ncbi.nlm.nih.gov/nuccore/OQ871366), OR189520 (*E. humifusa*, https://www.ncbi.nlm.nih.gov/nuccore/OR189520), and OR189521 (*E. maculate*; https://www.ncbi.nlm.nih.gov/nuccore/OR189521).
