# Supplementary material for: Characterization, comparison, and phylogenetic analyses of chloroplast genomes of Euphorbia species
Source: Sci Rep. 2024 Jul 4;14:15352. doi: 10.1038/s41598-024-66102-0 (PMC11222452; doi:10.1038/s41598-024-66102-0)
Supplement: Supplementary file 1 — Supplementary Information. [file 41598_2024_66102_MOESM1_ESM.docx]

Scientific Reports

**Supplementary Information**

Characterization of complete chloroplast genomes from nine *Euphorbia* species: Comparative, phylogenetic analyses and intraspecific variation

Soo-Rang Lee*, Ami Oh, and Dong-Chan Son*


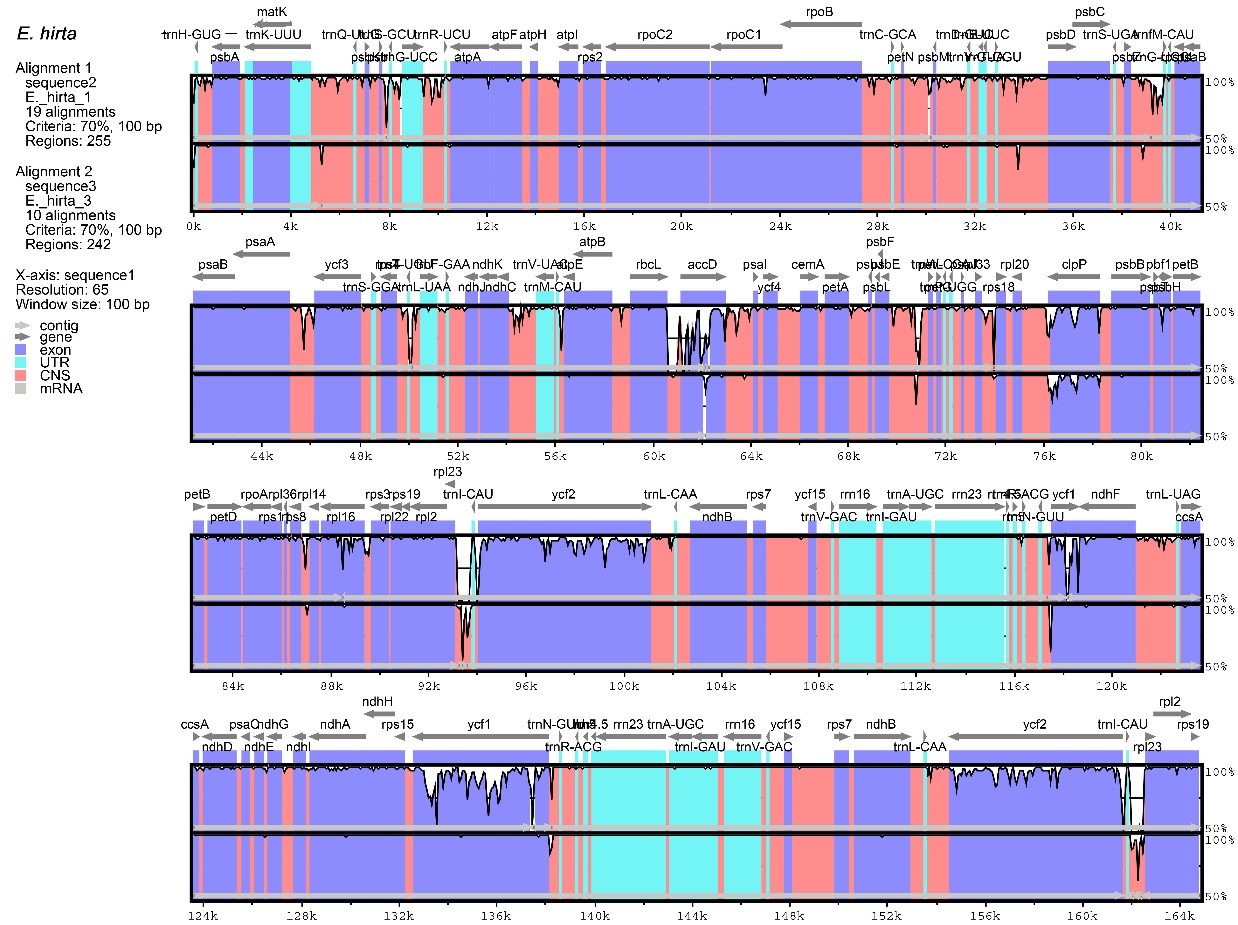


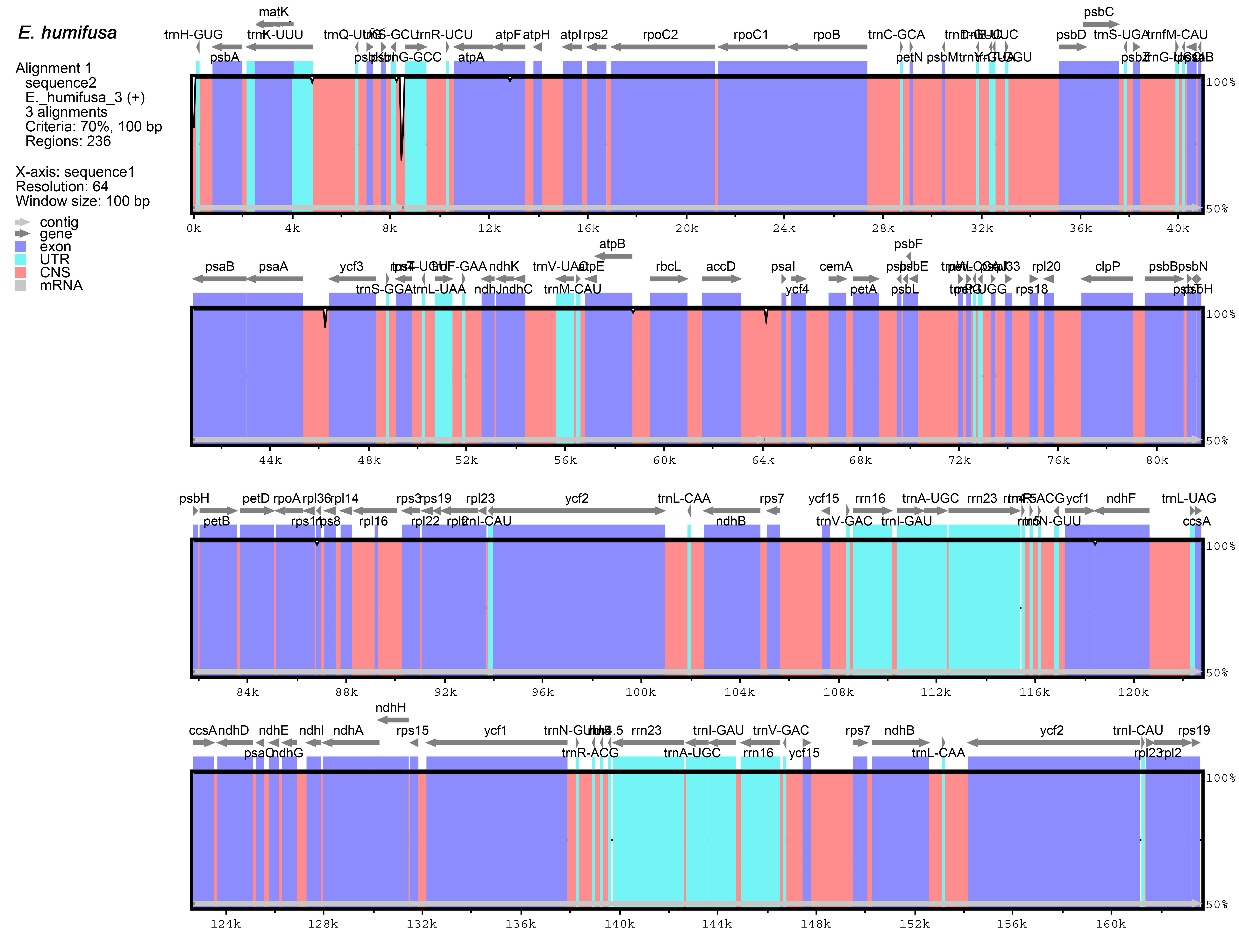


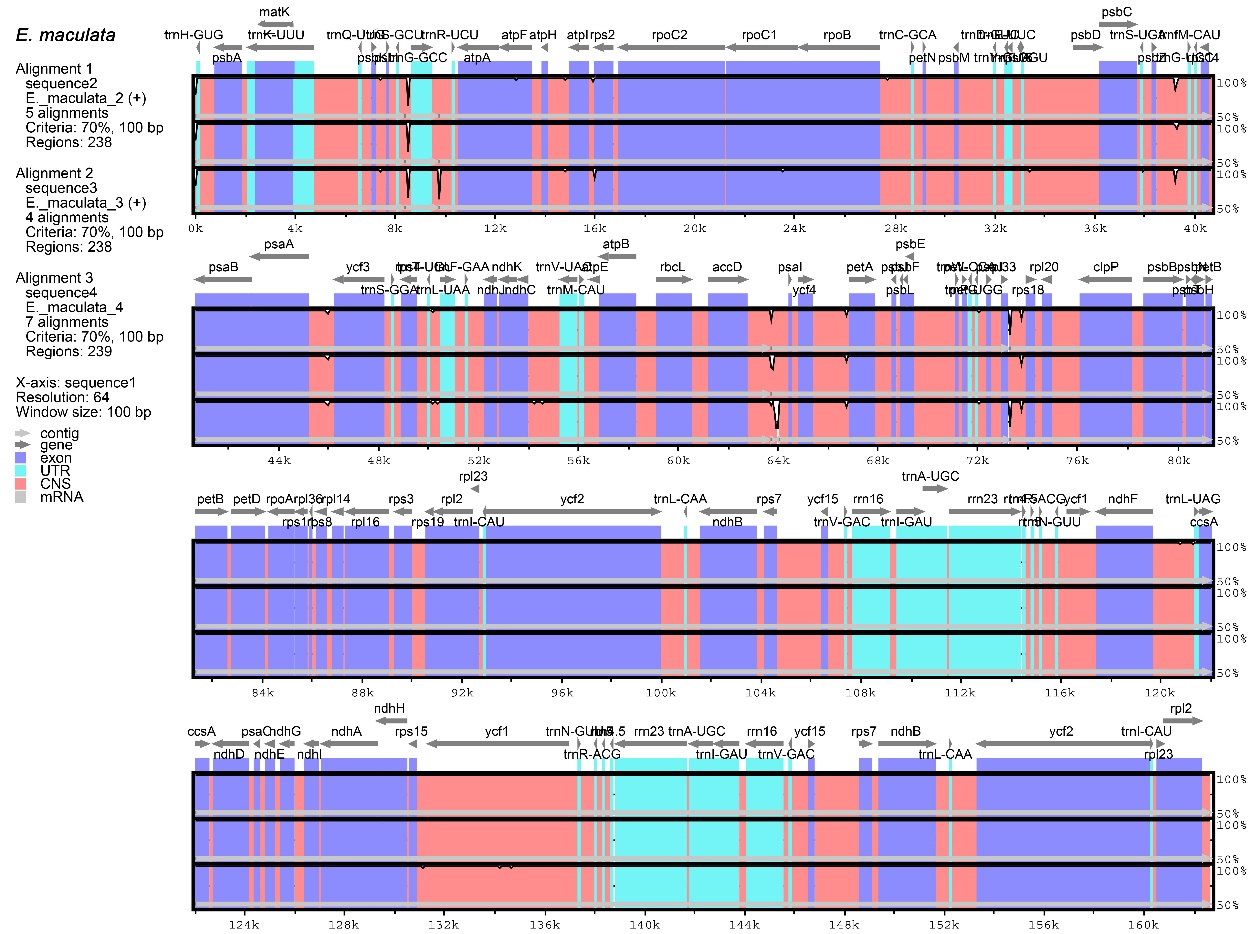


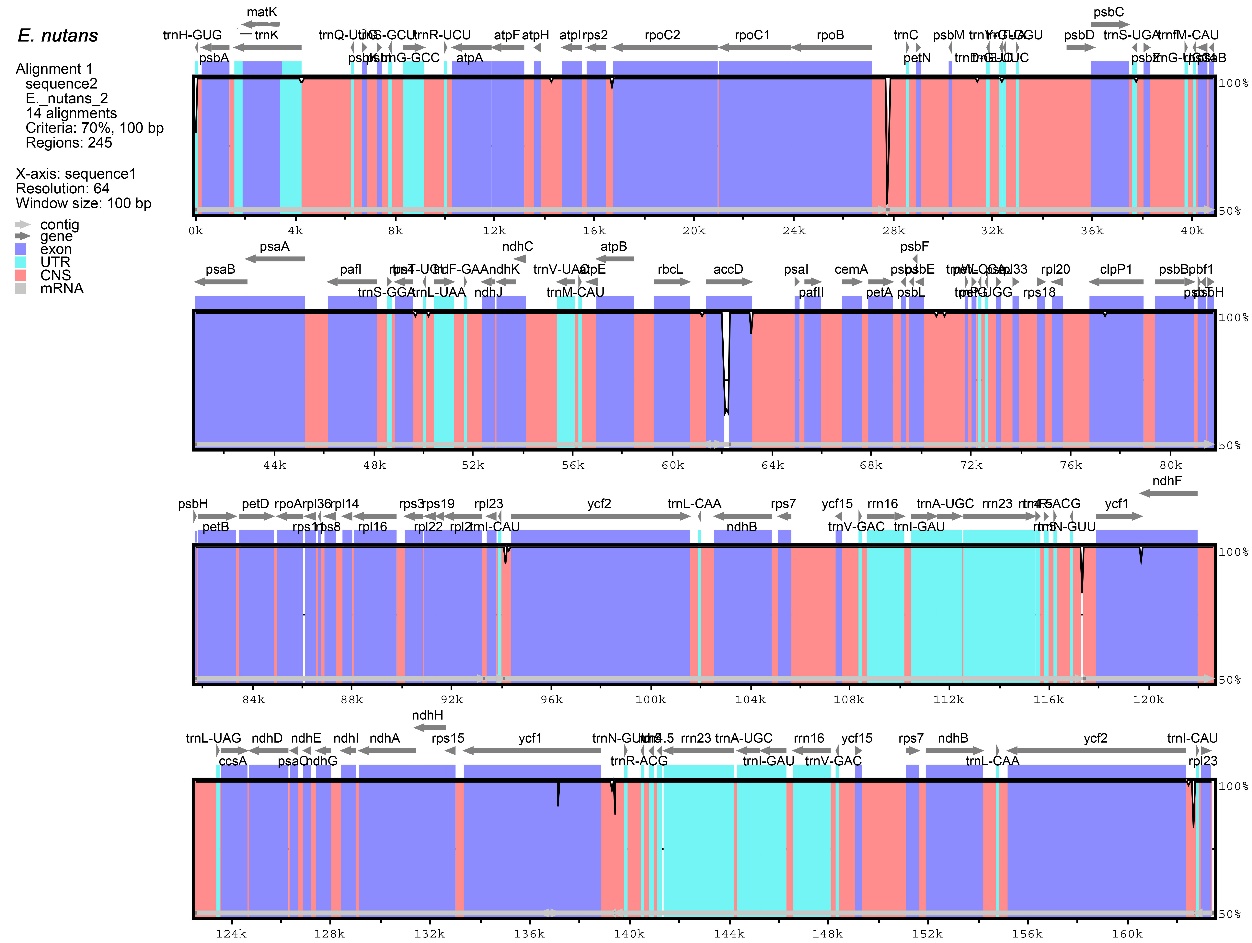


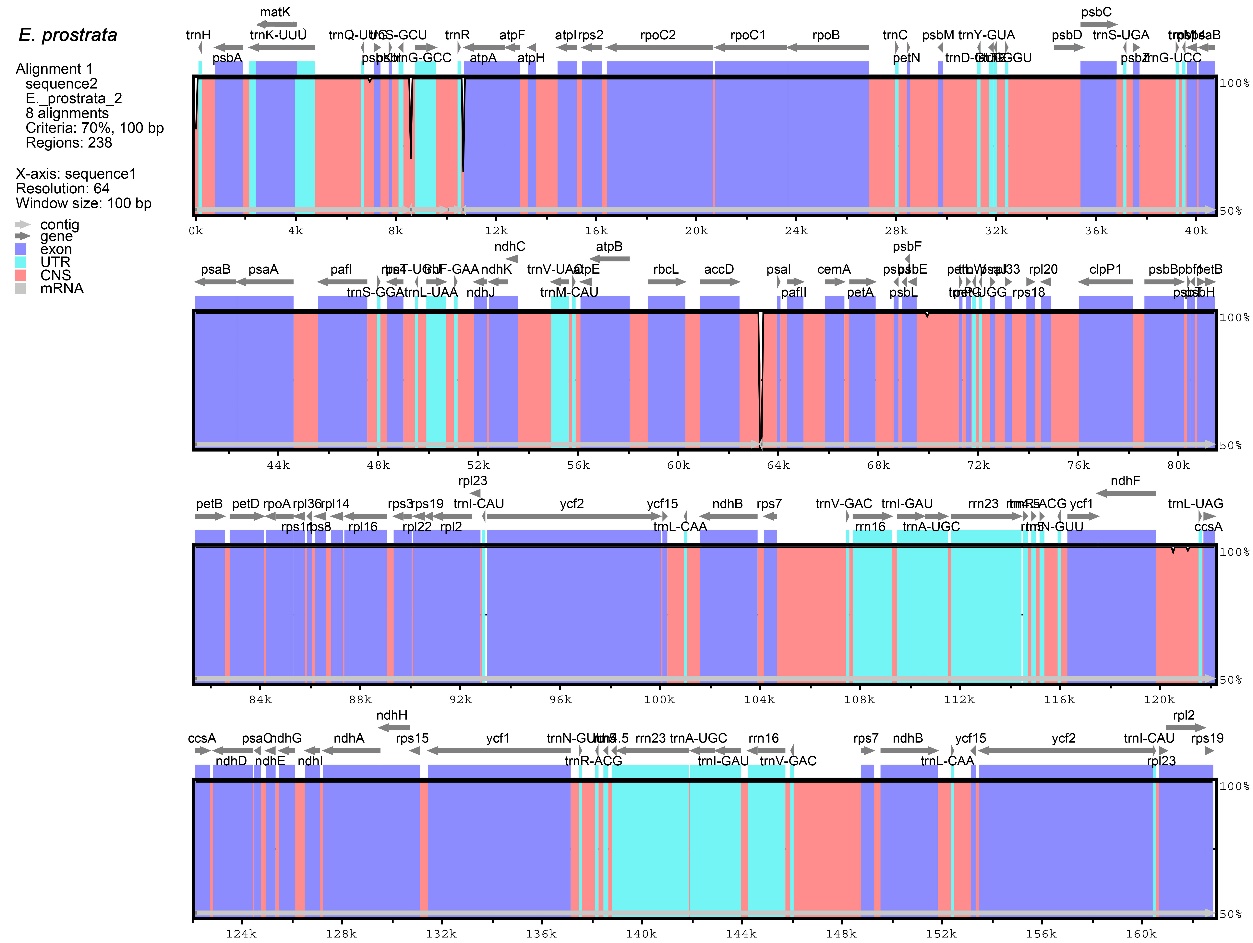


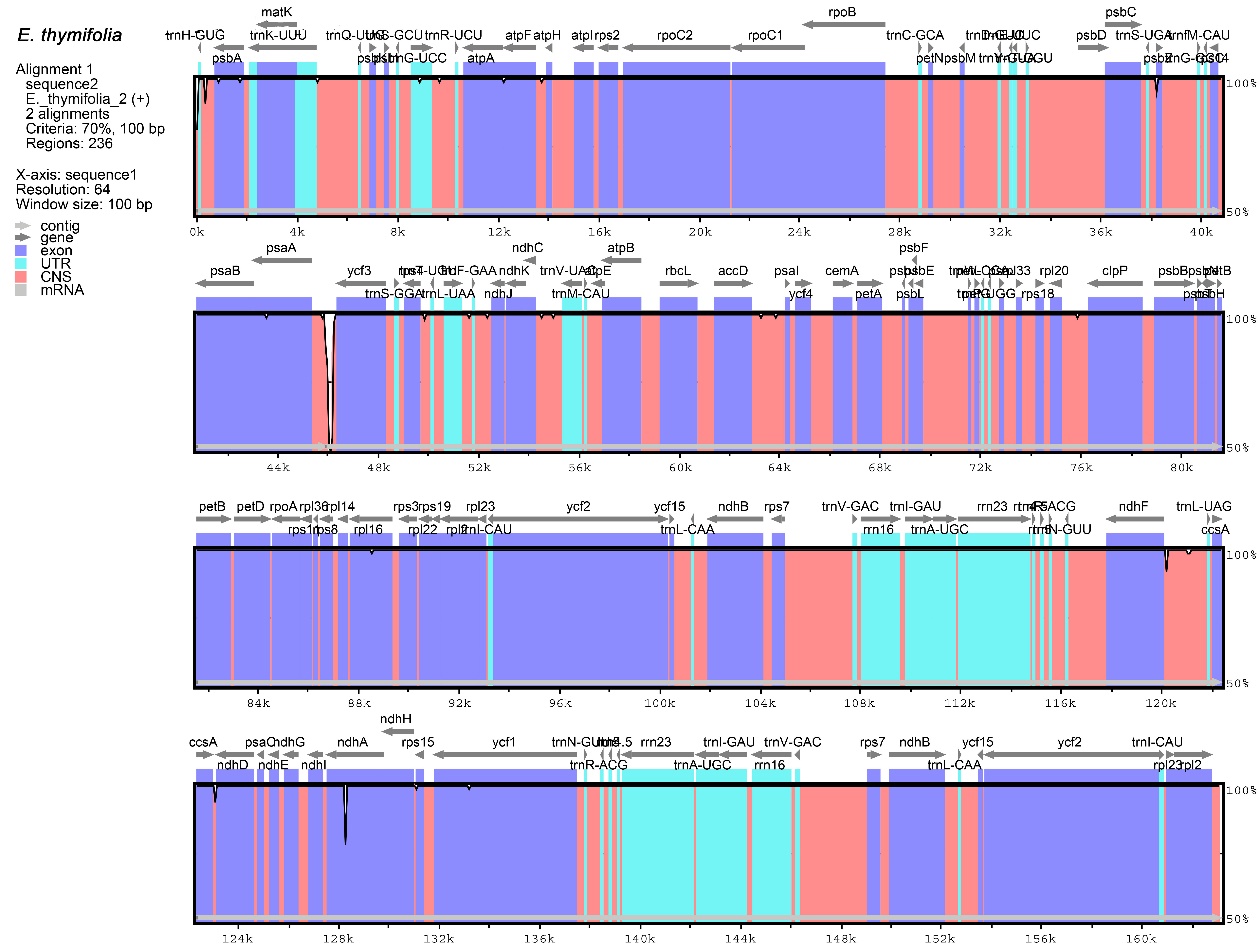


**Figure S1**. Summary plot depicting sequence divergence in chloroplast genomes among all accessions within each of the six *Euphorbia* species, estimated using mVISTA, with one accession serving as a reference. The Y-axis represents percent identity (ranging from 50% to 100%). Non-coding regions are shaded in pink, while coding regions are shaded in purple.


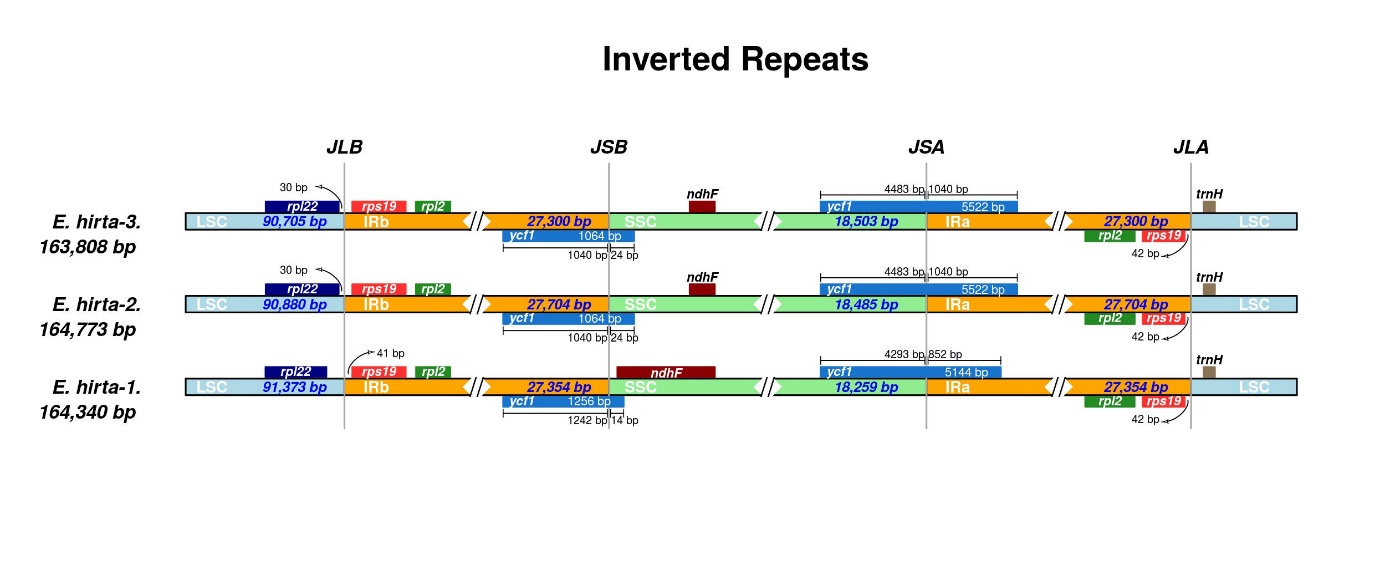

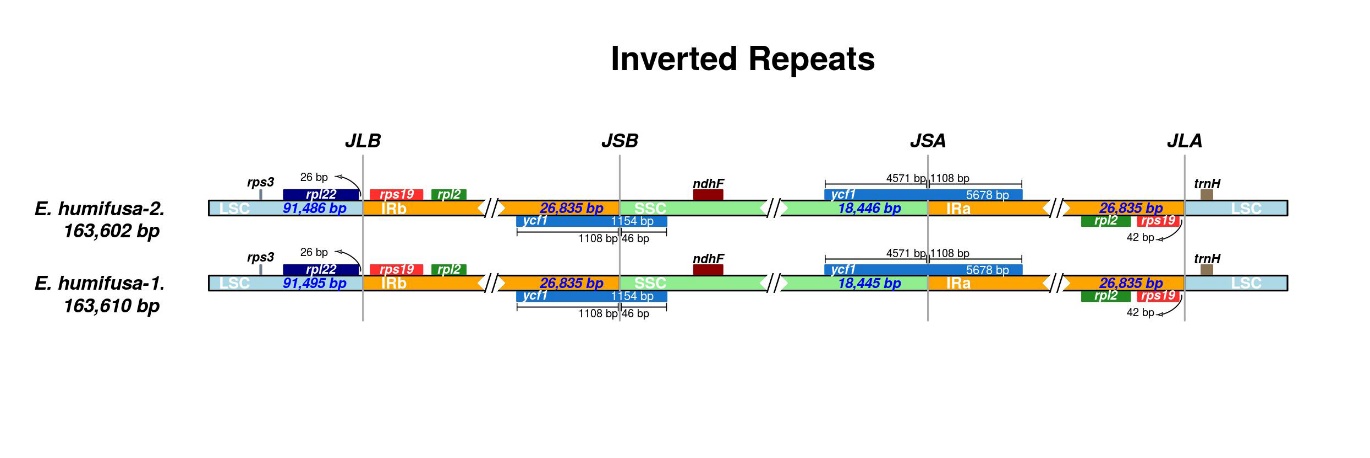

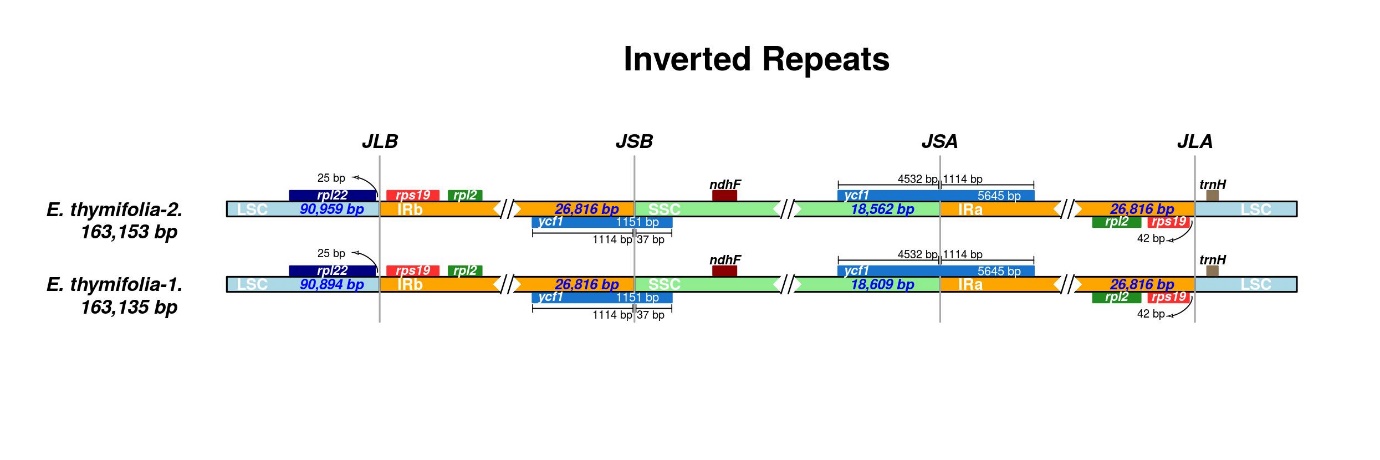

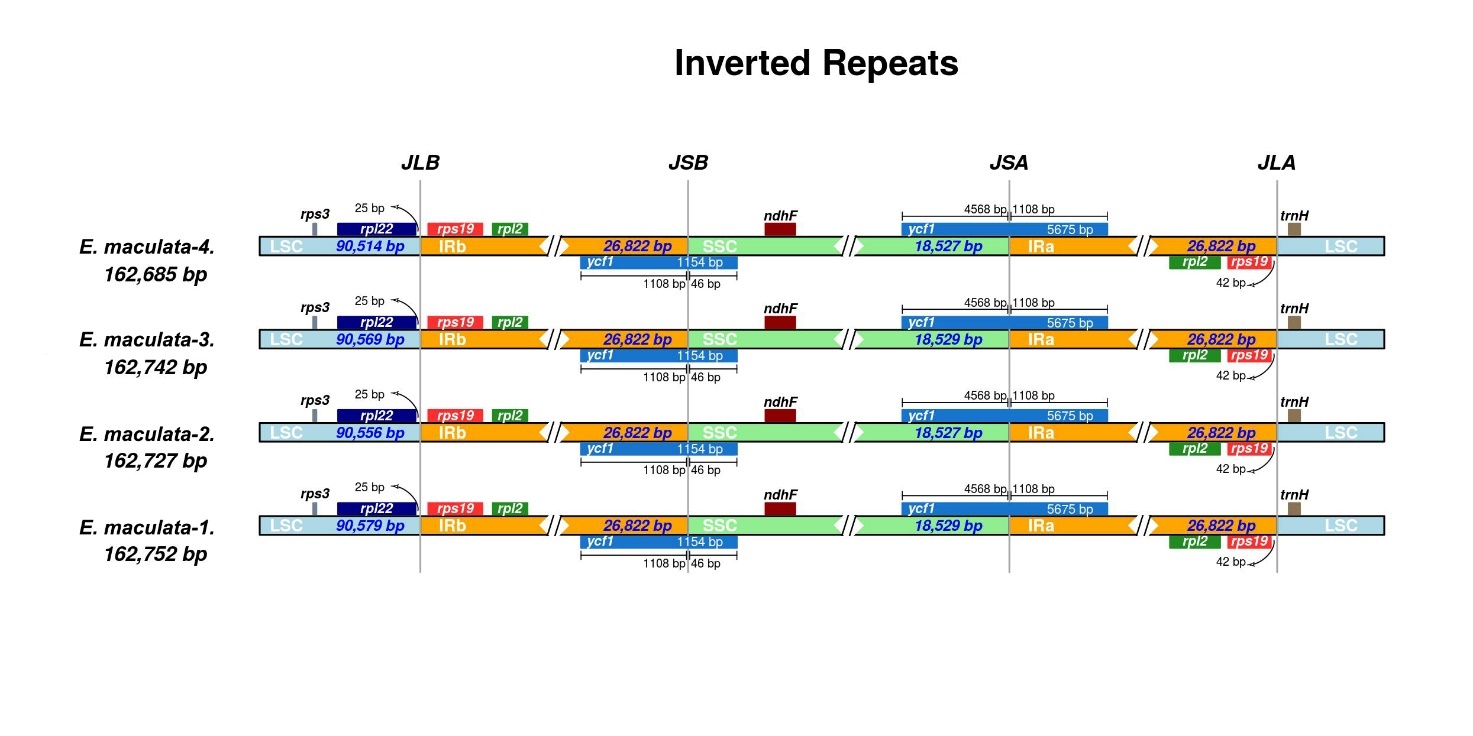

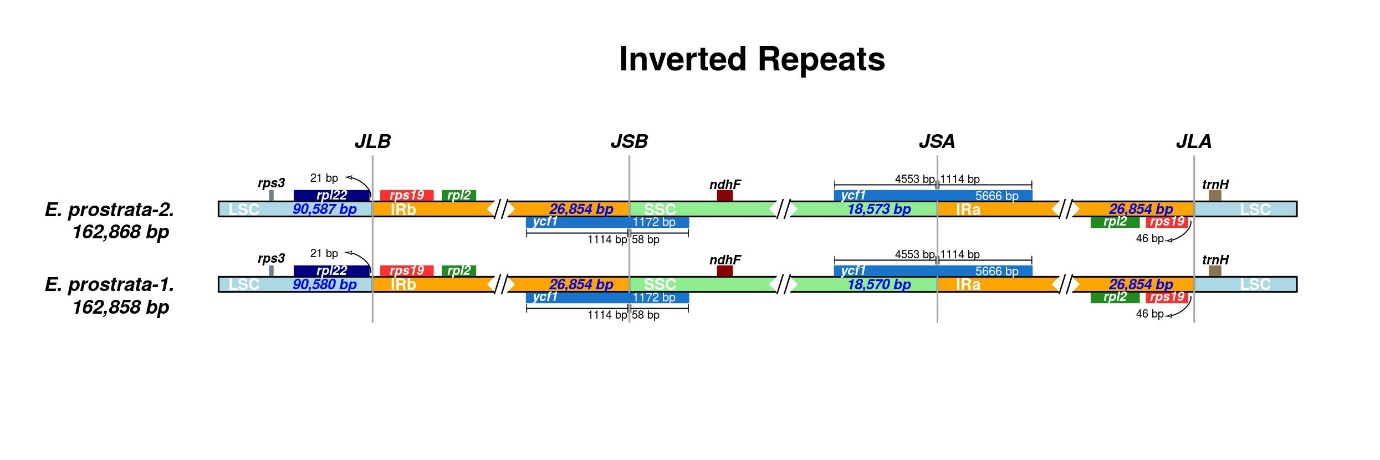

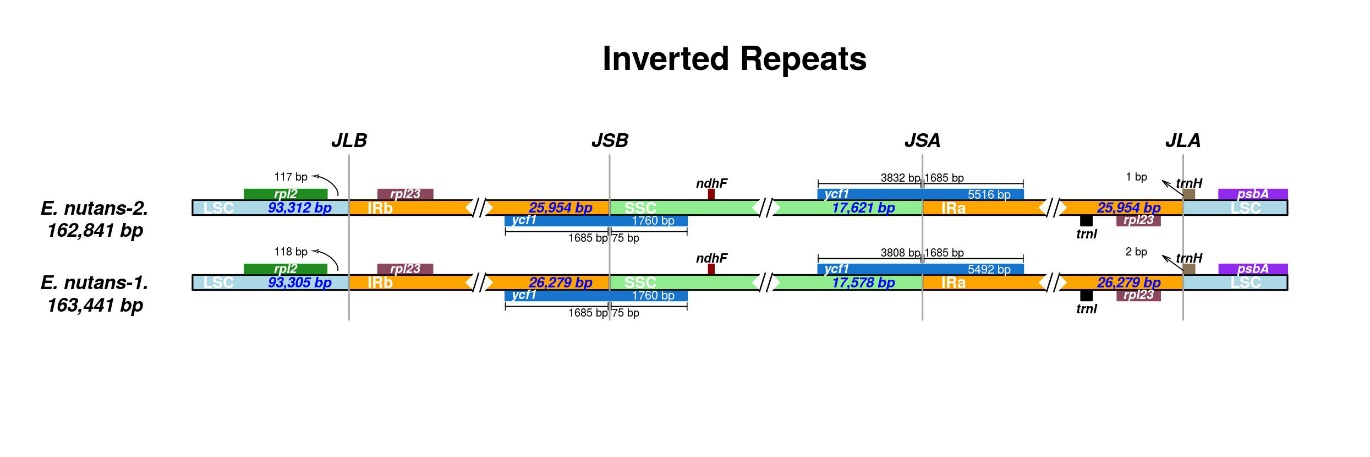


**Figure S2**. Comparison of the borders between the Large Single Copy (LSC), Inverted Repeat (IR), and Small Single Copy (SSC) regions in the chloroplast genomes among the available accessions within each *Euphorbia* species.


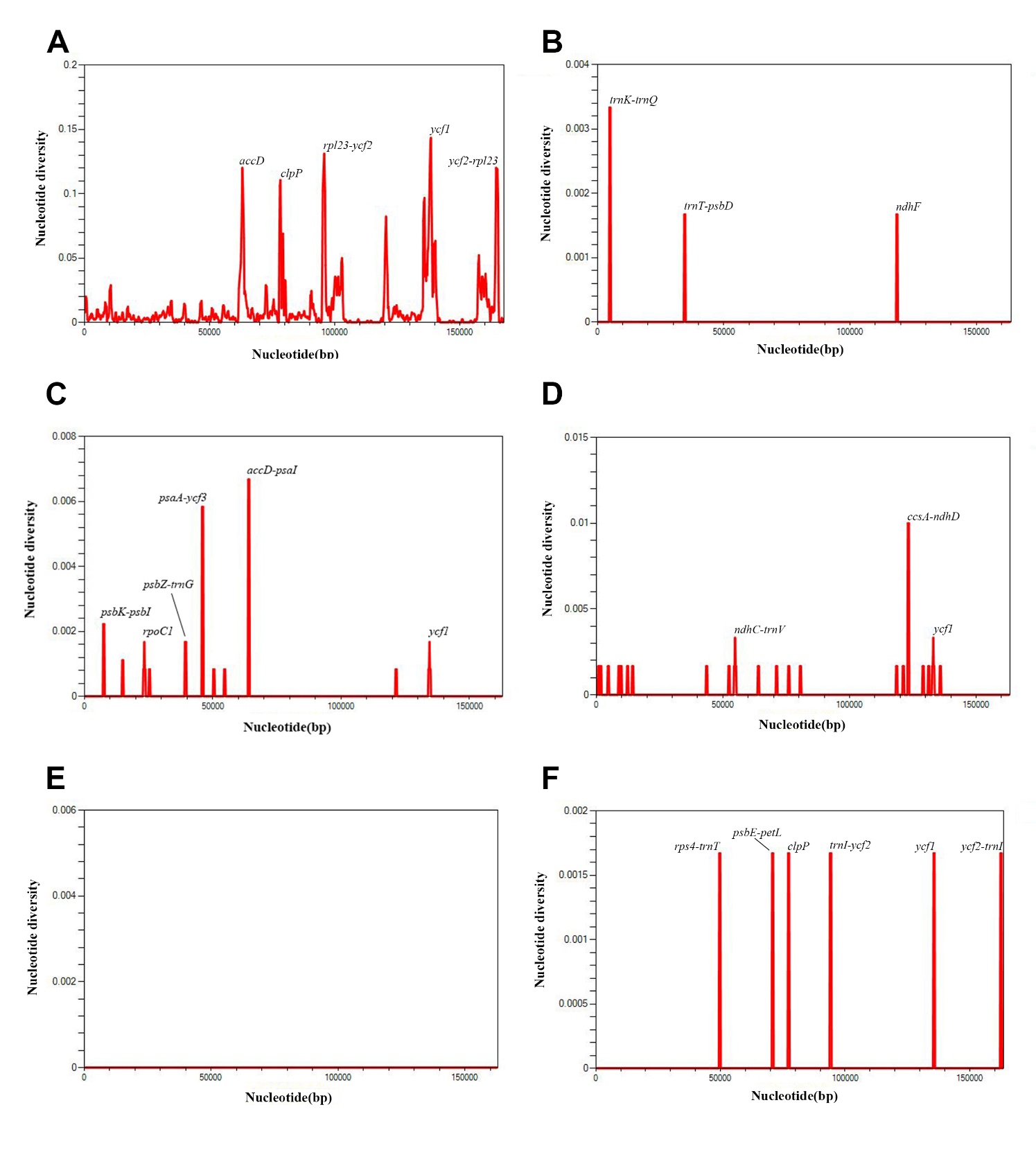


**Figure S3**. Nucleotide diversity in the CP genomes for all available accessions within each species of *Euphorbia*. (A) *E. hirta*. (B) *E. humifusa*. (C) *E. maculata*. (D) *E. thymifolia*. (E) *E. prostrata*. (F) *E. nutans*.


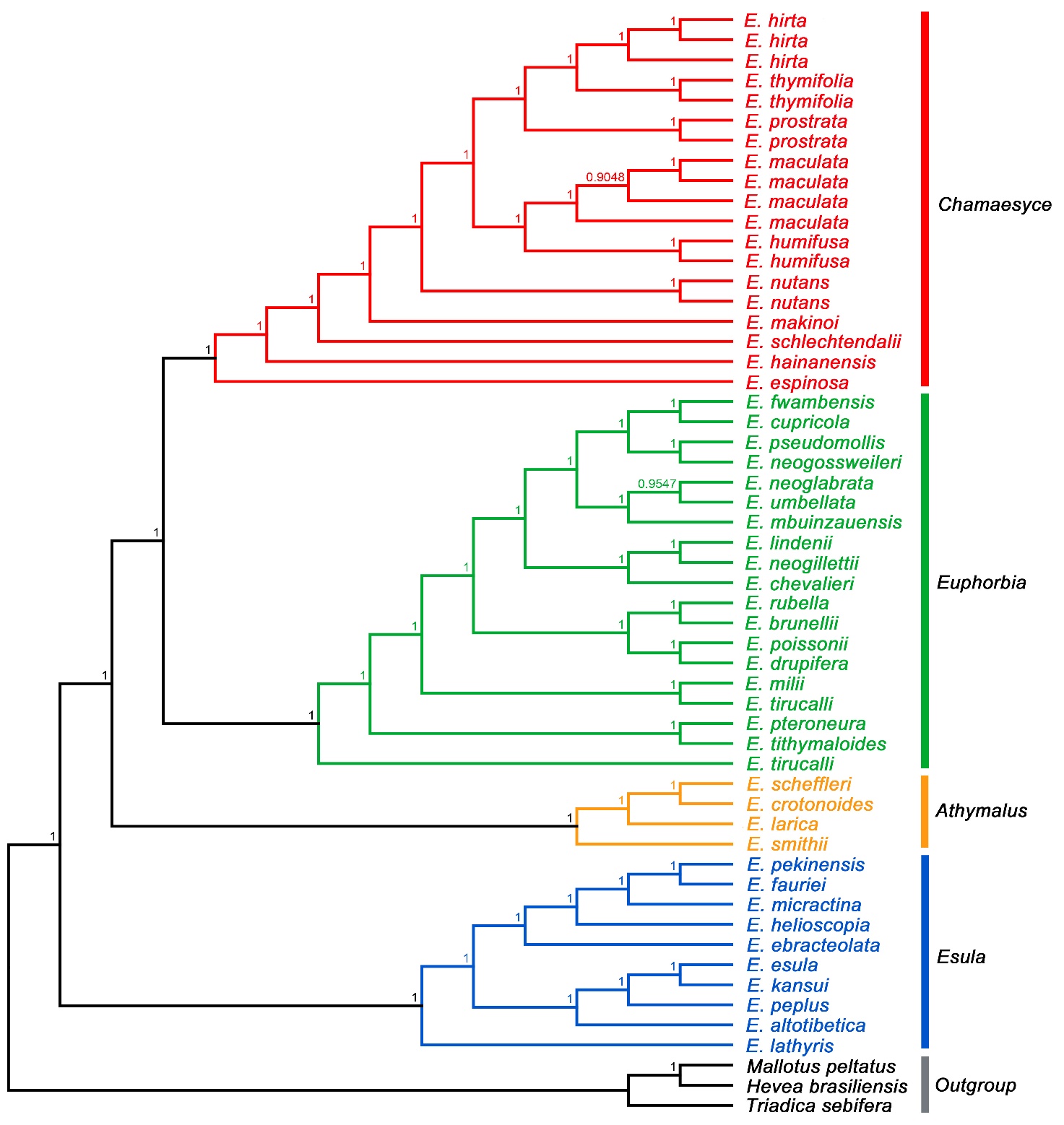


**Figure S4**. Phylogenetic relationships among 42 *Euphorbia* species inferred from complete CP genomes using the Bayesian Inference (BI) method. Posterior probability values are indicated at branch nodes. The four colors represent each of the four subgenera in *Euphorbia*.


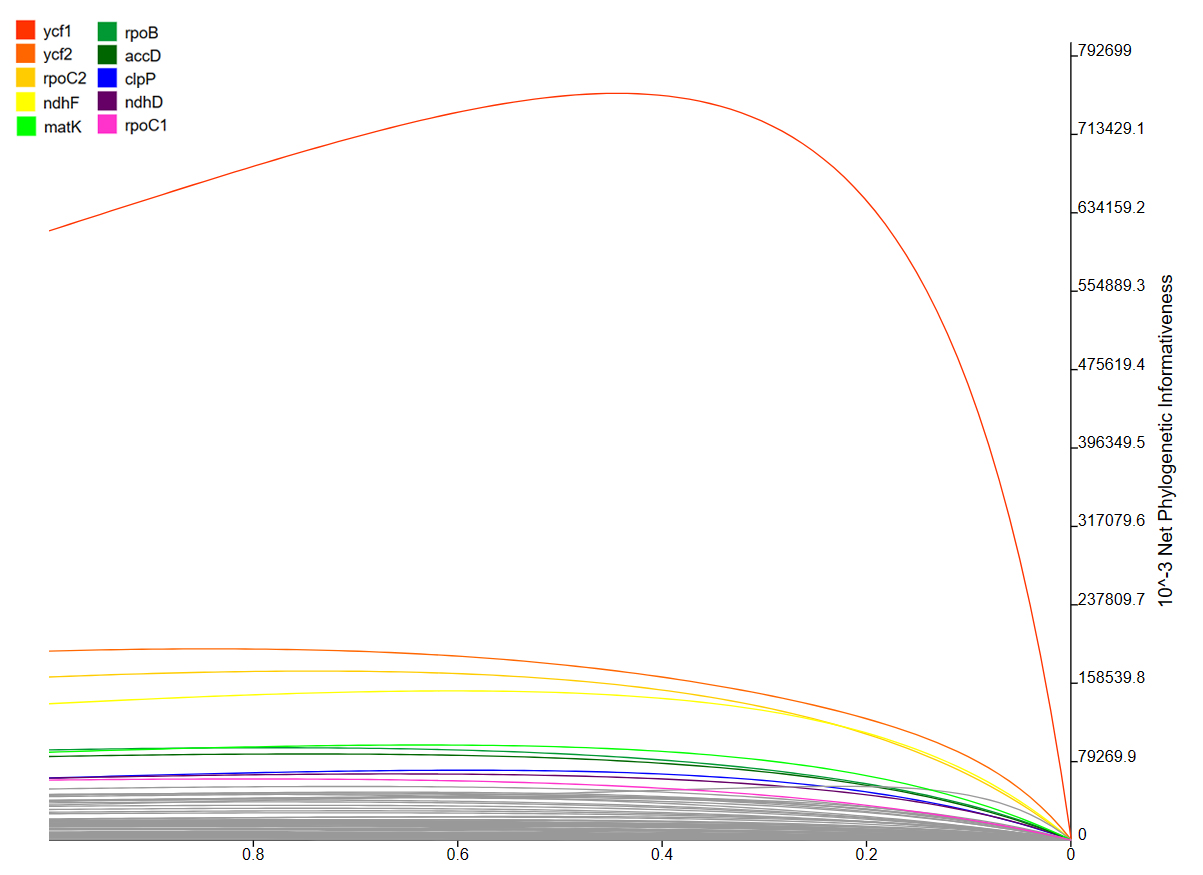


**Figure S5**. Phylogenetic informativeness (PI) net profiles for each coding sequence (CDS) on the chloroplast genome of *Euphorbia*, generated with PhyDesign. The colors correspond to each coding sequence with the highest PI values.

**Table S1**. List of accessions used for various analyses in the study. Accession numbers marked in bold are those that were checked and corrected in the study.

| Species name | GenBank Accession no. | | | mVISTA (interspecific) | | mVISTA (intraspecific) | | IRscope (interspecific) | | IRscope (intraspecific) | | pi calculation (interspecific) | | pi calculation (intraspecific) | | Genome size variation comparison | | Phylogeny (complete CP genome) | | Phylogeny (*rpoB*) |
| --- | --- | --- | --- | --- | --- | --- | --- | --- | --- | --- | --- | --- | --- | --- | --- | --- | --- | --- | --- | --- |
| *E. hirta*-1 | | **NC_058203** |  | | ○ | |  | | ○ | | ○ | | ○ | | ○ | | ○ | | ○ | |
| *E. hirta*-2 | | **MW429224** | ○ | | ○ | | ○ | | ○ | |  | | ○ | | ○ | | ○ | | ○ | |
| *E. hirta*-3 | | **OQ184032** |  | | ○ | |  | | ○ | |  | | ○ | | ○ | | ○ | | ○ | |
| *E. humifusa*-1 | | **OR189520** | ○ | | ○ | | ○ | | ○ | | ○ | | ○ | | ○ | | ○ | | ○ | |
| *E. humifusa*-2 | | **OQ184028** |  | | ○ | |  | | ○ | |  | | ○ | | ○ | | ○ | | ○ | |
| *E. maculata*-1 | | **NC_052745** | ○ | | ○ | | ○ | | ○ | | ○ | | ○ | | ○ | | ○ | | ○ | |
| *E. maculata*-2 | | **OR189521** |  | | ○ | |  | | ○ | |  | | ○ | | ○ | | ○ | | ○ | |
| *E. maculata*-3 | | **OQ184027** |  | | ○ | |  | | ○ | |  | | ○ | | ○ | | ○ | | ○ | |
| *E. maculata*-4 | | **MW496381** |  | | ○ | |  | | ○ | |  | | ○ | | ○ | | ○ | | ○ | |
| *E. thymifolia*-1 | | **NC_062827** | ○ | | ○ | | ○ | | ○ | | ○ | | ○ | | ○ | | ○ | | ○ | |
| *E. thymifolia*-2 | | **OQ184030** |  | | ○ | |  | | ○ | |  | | ○ | | ○ | | ○ | | ○ | |
| *E. prostrata*-1 | | **ON631059** | ○ | | ○ | | ○ | | ○ | | ○ | | ○ | | ○ | | ○ | | ○ | |
| *E. prostrata*-2 | | **OQ184029** |  | | ○ | |  | | ○ | |  | | ○ | | ○ | | ○ | | ○ | |
| *E. nutans*-1 | | **OQ871366** | ○ | | ○ | | ○ | | ○ | | ○ | | ○ | | ○ | | ○ | | ○ | |
| *E. nutans*-2 | | **NC_072939** |  | | ○ | |  | | ○ | |  | | ○ | | ○ | | ○ | | ○ | |
| *E. tirucalli* | | **NC_042193** | ○ | |  | | ○ | |  | | ○ | |  | | ○ | | ○ | | ○ | |
| *E. tirucalli* | | MT395048 |  | |  | |  | |  | |  | |  | |  | | ○ | | ○ | |
| *E. smithii* | | **MN646684** | ○ | |  | | ○ | |  | | ○ | |  | | ○ | | ○ | | ○ | |
| *E. pekinensis* | | **NC_058897** | ○ | |  | | ○ | |  | | ○ | |  | | ○ | | ○ | | ○ | |
| *E. makinoi* | | NC_072937 |  | |  | |  | |  | |  | |  | |  | | ○ | | ○ | |
| *E. schlechtendalii* | | NC_062826 |  | |  | |  | |  | |  | |  | |  | | ○ | | ○ | |
| *E. hainanensis* | | MH049548 |  | |  | |  | |  | |  | |  | |  | | ○ | |  | |
| *E. espinosa* | | MW496384 |  | |  | |  | |  | |  | |  | |  | | ○ | | ○ | |
| *E. fwambensis* | | MT528641 |  | |  | |  | |  | |  | |  | |  | | ○ | | ○ | |
| *E. cupricola* | | MW300677 |  | |  | |  | |  | |  | |  | |  | | ○ | | ○ | |
| *E. pseudomollis* | | MW300680 |  | |  | |  | |  | |  | |  | |  | | ○ | | ○ | |
| *E. neogossweileri* | | MT395027 |  | |  | |  | |  | |  | |  | |  | | ○ | | ○ | |
| *E. neoglabrata* | | MT394996 |  | |  | |  | |  | |  | |  | |  | | ○ | | ○ | |
| *E. umbellata* | | MT395046 |  | |  | |  | |  | |  | |  | |  | | ○ | | ○ | |
| *E. mbuinzauensis* | | MT395000 |  | |  | |  | |  | |  | |  | |  | | ○ | | ○ | |
| *E. lindenii* | | MT395026 |  | |  | |  | |  | |  | |  | |  | | ○ | | ○ | |
| *E. neogillettii* | | MT395021 |  | |  | |  | |  | |  | |  | |  | | ○ | | ○ | |
| *E. chevalieri* | | MT528649 |  | |  | |  | |  | |  | |  | |  | | ○ | | ○ | |
| *E. rubella* | | MT528639 |  | |  | |  | |  | |  | |  | |  | | ○ | | ○ | |
| *E. brunellii* | | MT528638 |  | |  | |  | |  | |  | |  | |  | | ○ | | ○ | |
| *E. poissonii* | | MT395035 |  | |  | |  | |  | |  | |  | |  | | ○ | | ○ | |
| *E. drupifera* | | MW496383 |  | |  | |  | |  | |  | |  | |  | | ○ | | ○ | |
| *E. milii* | | MW496385 |  | |  | |  | |  | |  | |  | |  | | ○ | | ○ | |
| *E. pteroneura* | | MW496386 |  | |  | |  | |  | |  | |  | |  | | ○ | | ○ | |
| *E. tithymaloides* | | MW496382 |  | |  | |  | |  | |  | |  | |  | | ○ | | ○ | |
| *E. scheffleri* | | MT395025 |  | |  | |  | |  | |  | |  | |  | | ○ | | ○ | |
| *E. crotonoides* | | MW496380 |  | |  | |  | |  | |  | |  | |  | | ○ | | ○ | |
| *E. larica* | | MN646683 |  | |  | |  | |  | |  | |  | |  | | ○ | | ○ | |
| *E. fauriei* | | NC_067640 |  | |  | |  | |  | |  | |  | |  | | ○ | | ○ | |
| *E. micractina* | | NC_060656 |  | |  | |  | |  | |  | |  | |  | | ○ | | ○ | |
| *E. helioscopia* | | MN199031 |  | |  | |  | |  | |  | |  | |  | | ○ | | ○ | |
| *E. ebracteolata* | | MT830860 |  | |  | |  | |  | |  | |  | |  | | ○ | | ○ | |
| *E. esula* | | KY000001 |  | |  | |  | |  | |  | |  | |  | | ○ | | ○ | |
| *E. kansui* | | MH392274 |  | |  | |  | |  | |  | |  | |  | | ○ | | ○ | |
| *E. peplus* | | MZ678242 |  | |  | |  | |  | |  | |  | |  | | ○ | | ○ | |
| *E. altotibetica* | | NC_066896 |  | |  | |  | |  | |  | |  | |  | | ○ | | ○ | |
| *E. lathyris* | | MT241376 |  | |  | |  | |  | |  | |  | |  | | ○ | | ○ | |
| *Mallotus peltatus* | | MN885802 |  | |  | |  | |  | |  | |  | |  | | ○ | | ○ | |
| *Hevea brasiliensis* | | HQ285842 |  | |  | |  | |  | |  | |  | |  | | ○ | | ○ | |
| *Triadica sebifera* | | MT424756 |  | |  | |  | |  | |  | |  | |  | | ○ | | ○ | |

**Table S2**. List of Tree Distance values calculated for genes with high nucleotide diversity (pi, 5 genes) and/or phylogenetic informativeness (PI, 10 genes). The last raw presents Tree Distance values of 7 different combinations of three candidate genes with lowest Tree Distance values. Genes in bold face refer to the top 3 genes showing the highest tree congruence with the lowest tree distance from the plastome tree.

| Category | Combination | Tree Distance |
| --- | --- | --- |
| High pi regions | *accD-psaI* | 0.2038646 |
|  | *ndhF-trnL* | 0.1887916 |
|  | *psbI-atpA* | 0.1275786 |
|  | *trnK-trnQ* | 0.1774569 |
|  | *ycf1* | 0.1277837 |
| High PI regions | *ycf1* | 0.1277837 |
|  | *accD* | 0.1871502 |
|  | *clpP* | 0.1782533 |
|  | *ycf2* | 0.145493 |
|  | *ndhD* | 0.1361845 |
|  | *ndhF* | 0.1111143 |
|  | *matK* | 0.1026027 |
|  | ***rpoC2*** | **0.1008332** |
|  | ***rpoC1*** | **0.09739315** |
|  | ***rpoB*** | **0.07906522** |
| 7 different combinations with top three genes | *rpoB* | 0.07906522 |
|  | *rpoC1* | 0.09739315 |
|  | *rpoC2* | 0.1008332 |
|  | *rpoB + rpoC1* | 0.09435442 |
|  | *rpoB + rpoC2* | 0.08486776 |
|  | *rpoC1 + rpoC2* | 0.09575126 |
|  | *rpoB + rpoC1 + rpoC2* | 0.0902543 |
